# A spatially aware likelihood test to detect sweeps from haplotype distributions

**DOI:** 10.1371/journal.pgen.1010134

**Published:** 2022-04-11

**Authors:** Michael DeGiorgio, Zachary A. Szpiech

**Affiliations:** 1 Department of Electrical Engineering and Computer Science, Florida Atlantic University, Boca Raton, Florida, United States of America; 2 Department of Biology, Pennsylvania State University, University Park, Pennsylvania, United States of America; 3 Institute for Computational and Data Sciences, Pennsylvania State University, University Park, Pennsylvania, United States of America; University of Wyoming, UNITED STATES

## Abstract

The inference of positive selection in genomes is a problem of great interest in evolutionary genomics. By identifying putative regions of the genome that contain adaptive mutations, we are able to learn about the biology of organisms and their evolutionary history. Here we introduce a composite likelihood method that identifies recently completed or ongoing positive selection by searching for extreme distortions in the spatial distribution of the haplotype frequency spectrum along the genome relative to the genome-wide expectation taken as neutrality. Furthermore, the method simultaneously infers two parameters of the sweep: the number of sweeping haplotypes and the “width” of the sweep, which is related to the strength and timing of selection. We demonstrate that this method outperforms the leading haplotype-based selection statistics, though strong signals in low-recombination regions merit extra scrutiny. As a positive control, we apply it to two well-studied human populations from the 1000 Genomes Project and examine haplotype frequency spectrum patterns at the *LCT* and MHC loci. We also apply it to a data set of brown rats sampled in NYC and identify genes related to olfactory perception. To facilitate use of this method, we have implemented it in user-friendly open source software.

## Author summary

Identifying regions of the genome that contain adaptive variation is of fundamental interest in evolutionary biology, providing insight into an organism’s history and biology. When positive selection is recent or ongoing, we expect to find genomic patterns such as high frequency haplotypes and low genetic diversity in the vicinity of the adaptive locus. Here we develop a statistic to identify these regions based on distortions of the haplotype frequency spectrum from a background distribution. We evaluate the performance of this statistic under numerous realistic settings of interest to empiricists and demonstrate its superior performance relative to other haplotype-based selection statistics. We also apply this statistic to real population-genetic data. As a positive control, we explore two well-studied loci, *LCT* and MHC, in a European and an African human population that show strong evidence for selection. We also apply this statistic to the genomes of an urban brown rat population, where we uncover evidence for adaptation in olfactory perception genes. We release user-friendly software implementing this statistic.

## Introduction

The identification and classification of genomic regions undergoing positive selection in populations has been of long standing interest for studying organisms across the tree of life. By investigating regions containing putative adaptive variation, one can begin to shed light on a population’s evolutionary history and the biological changes well-suited to cope with various selection pressures.

The genomic footprint of positive selection is generally characterized by long high-frequency haplotypes and low nucleotide diversity in the vicinity of the adaptive locus, the result of linked genetic material “sweeping” to high frequency faster than mutation and recombination can introduce novel variation. These selective sweeps are often described by two paradigms—“hard sweeps” and “soft sweeps”. Whereas a hard sweep is the result of a beneficial mutation that brings a single haplotype to high frequency [1], soft sweeps are the result of selection on multiple haplotype backgrounds, often the result of selection on standing variation or a high adaptive mutation rate. Soft sweeps are thus characterized by multiple sweeping haplotypes rising to high frequency [2, 3].

Many statistics have been proposed to capture these patterns to make inferences about recent or ongoing positive selection [4–24], many of which focus on summarizing patterns of haplotype homozygosity in a local genomic region. A particularly novel approach, the *T* statistic implemented in LASSI [13], employs a likelihood model based on distortions of the haplotype frequency spectrum (HFS). In this framework, [13] model a shift in the HFS toward one or several high-frequency haplotypes as the result of a hard or soft sweep in a local region of the genome. In addition to the likelihood test statistic *T*, for which larger values suggest more support for a sweep, LASSI also infers the parameter $\hat{m}$. This parameter estimates the number of sweeping haplotypes in a genomic region, and $\hat{m}>1$ indicates support for a soft sweep.

A drawback of the original formulation of the *T* statistic implemented in LASSI is that it does not account for or make use of the genomic spatial distribution of haplotypic variation expected from a sweep. Specifically, [13] demonstrated that if the spatial distribution of *T* was directly accounted for in the machine learning approach (*Trendsetter*) of [25], the power for detecting sweeps was greatly enhanced. Indeed, modern statistical learning machinery to detect sweeps has been greatly enhanced by incorporating spatial distributions of summary statistics [25–30]. However, these machine learning methods need extensive simulations under an accurate and explicit demographic model to train the classifier. An alternative approach is to directly integrate this spatial distribution into the likelihood model, as has been performed for site frequency spectrum (SFS) composite likelihood methods to detect sweeps [16–24]. Here we incorporate the spatial distribution along the genome of HFS variation into the LASSI framework and introduce the Spatially Aware Likelihood Test for Improving LASSI, or saltiLASSI. For easy application to genomic datasets, we implement saltiLASSI in the open source program lassip along with LASSI [13], and other HFS-based statistics H12, H2/H1, G123, and G2/G1 [8, 10]. lassip is available at https://www.github.com/szpiech/lassip.

We validate saltiLASSI through simulations and compare it favorably to other popular haplotype-based selection scans. As this is a composite likelihood statistic, it is likely to be affected by recombination rate variation, and we therefore explore strategies for estimating the statistic’s variance under neutrality in this context. We note that, in general, strong signals found in low-recombination regions should be treated with extra scrutiny. We next apply saltiLASSI to whole genome data from two different species. These data include two well-studied human populations (CEU and YRI) from the 1000 Genomes Project [31] and a population of brown rats sampled across the island of Manhattan in New York City (NYC), USA [32]. Our analysis of the two human populations serves as a positive control in an empirical dataset with a well-studied demographic history. We reproduce several well-known signals of selection in the European CEU population and the African YRI population, including the *LCT* (CEU), MHC (CEU and YRI), and *APOL1* (YRI) loci, demonstrating that this method works well in real data. Our analysis of the NYC brown rat data serves as an example of applying the saltiLASSI method to a dataset with haplotype phase unknown and a poorly calibrated demographic history making neutral simulations contraindicated (see [32] on this point). Here, we find strong selection signals among clusters of genes related to olfactory perception.

## Results

In this section we begin by developing a new likelihood ratio test statistic, termed Λ, that evaluates spatial patterns in the distortion of the HFS as evidence for sweeps. We then demonstrate that Λ has substantially higher power than competing single-population haplotype-based approaches, across a number of model parameters related to the underlying demographic and adaptive processes. Similar to the *T* statistic implemented in the LASSI framework of [13], we also show that Λ is capable of approximating the softness of a sweep by estimating the current number of high-frequency haplotypes $\hat{m}$. We then apply the Λ statistic to whole-genome sequencing data from two human populations from the 1000 Genomes Project [31] and a population of brown rats from NYC [32].

### Definition of the statistic

**Fig 1:**
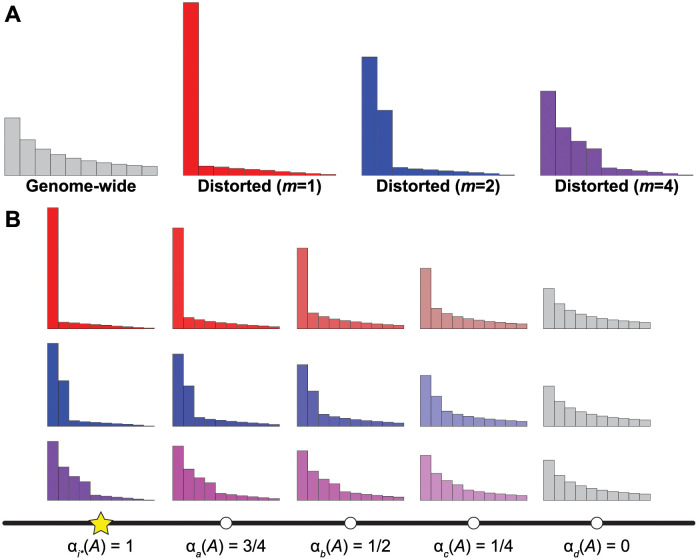
Schematic of the saltiLASSI mixture model framework. (A) Generation of distorted haplotype frequency spectra (HFS) for *m* = 1 (red), 2 (blue), and 4 (purple) sweeping haplotypes from a genome-wide (gray) neutral HFS under the LASSI framework of [13]. (B) Generation of spatially-distorted HFS under the saltiLASSI framework for a window *i* (white circles) with increasing distance from the sweep location (yellow star). When the window is on top of the sweep location, the HFS is identical to the distorted LASSI HFS, and *α*_*i*_(*A*) = 1. When a window is far from the sweep location, the HFS is identical to the genome-wide (neutral) HFS, and *α*_*i*^⋆^_ (*A*) = 0. For windows at intermediate distances from the sweep location, the HFS is a mixture of the distorted and genome-wide HFS, with the distorted HFS contributing *α*_*i*_(*A*) and the genome-wide HFS contributing 1 − *α*_*i*_(*A*). We show example spectra at windows *a*, *b*, *c*, and *d* that are of increasing distances from the sweep location *i*^⋆^, with *i*^⋆^ < *a* < *b* < *c* < *d*.

Here we extend the LASSI maximum likelihood framework for detecting sweeps based on haplotype data [13], by incorporating the spatial pattern of haplotype frequency distortion in a statistical model of a sweep. Recall that [13] defined a genome-wide background *K*-haplotype truncated frequency spectrum vector

$$\begin{array}{c} p=(p_{1},p_{2},\ldots ,p_{K}), \end{array}$$

which they assume represents the neutral distribution of the *K* most-frequent haplotypes, with *p*_1_ ≥ *p*_2_ ≥ ⋯ ≥ *p*_*K*_ ≥ 0 and normalization such that $\sum_{k=1}^{K}p_{k}=1$. [13] then define the vector

$$\begin{array}{c} q^{\left(m\right)}=(q_{1}^{\left(m\right)},q_{2}^{\left(m\right)},\ldots ,q_{K}^{\left(m\right)}), \end{array}$$

with $q_{1}^{\left(m\right)}\geq q_{2}^{\left(m\right)}\geq \cdots \geq q_{K}^{\left(m\right)}$ and $\sum_{k=1}^{K}q_{k}^{\left(m\right)}=1$. This represents a distorted *K*-haplotype truncated frequency spectrum vector in a particular genomic region with a distortion consistent with *m* sweeping haplotypes. To create the these distorted haplotype spectra, [13] used the equation

$$\begin{array}{c} q_{k}^{\left(m\right)}=\{\begin{array}{cc} p_{k}+f_{k}\sum_{j=m+1}^{K}\left(p_{j}-q_{j}^{\left(m\right)}\right) & k=1,2,\ldots ,m\\ U-\frac{k-m-1}{K-m-1}\left(U-\varepsilon \right) & k=m+1,m+2,\ldots ,K \end{array} \end{array}$$

where *f*_*k*_ ≥ 0 for *k* ∈ {1, 2, …, *m*} and $\sum_{k=1}^{m}f_{k}=1$, defines the way by which mass is distributed to the *m* “sweeping” haplotypes from the *K* − *m* non-sweeping haplotypes with frequencies *p*_*m*+1_, *p*_*m*+2_, …, *p*_*K*_. The variables *U* and *ε* are associated with the amount of mass from non-sweeping haplotypes that are converted to the *m* sweeping haplotypes (see [13]). We choose to set *U* = *p*_*K*_, and then vary *ε* ≤ *U* during optimization. [13] propose several reasonable choices of *f*_*k*_, and for all computations here we use $f_{k}=e^{-k}/\sum_{j=1}^{m}e^{-j}$. The schematic in Fig 1A illustrates the LASSI framework of generating the distorted haplotype spectra.

To incorporate the spatial distribution haplotypic variation into the LASSI framework, consider an index set W={1,2,…,I} of $I\in Z^{+}$ contiguous (potentially overlapping) windows such that window i∈W has position along a chromosome denoted *z*_*i*_. This position could be in physical units (such as bases), in genetic map units (such as centiMorgans), in number of polymorphic sites (such as employed by *nS*_*L*_ in [7]), or in window number. We model the relative contribution of a sweep with *m* sweeping haplotypes at target window with index $i^{⋆}\in W$ by a parameter *α*_*i*_ ∈ [0, 1] on window i∈W and the relative contribution of neutrality by 1 − *α*_*i*_.

Following a similar powerful framework introduced by [33] for modeling balancing selection, we employ a mixture model to model the *K*-haplotype truncated frequency spectrum in window *i*, with a proportion

$$\begin{array}{c} \alpha_{i}\left(A\right)=\text{exp}(-A|z_{i}-z_{i^{⋆}}|) \end{array}$$

deriving from a sweep model and a proportion 1 − *α*_*i*_(*A*) deriving from the genome-wide background haplotype spectrum to represent neutrality. Here, *A* is a parameter that we optimize over, describing the rate of decay of the effect of the sweep at target window *i*^⋆^ on the flanking windows a certain distance away. Specifically, we model the *K*-truncated haplotype spectrum in window *i* as the vector

$$\begin{array}{c} g_{i}^{\left(m,A\right)}=(g_{i1}^{\left(m,A\right)},g_{i2}^{\left(m,A\right)},\ldots ,g_{iK}^{\left(m,A\right)}), \end{array}$$

where

$$\begin{array}{c} g_{ik}^{\left(m,A\right)}=\alpha_{i}\left(A\right)q_{k}^{\left(m\right)}+\left[1-\alpha_{i}\left(A\right)\right]p_{k} \end{array}$$

for *k* = 1, 2, …, *K* and i∈W. Note here that for target window *i*^⋆^, $\alpha_{i^{⋆}}(A)=1$, and hence $g_{i^{⋆}}^{\left(m,A\right)}=q_{i^{⋆}}^{\left(m\right)}$—*i.e*., the target window is on top of the sweep, and so it is entirely determined by the distorted *m*-sweeping haplotype spectrum. However given a fixed *A* value, for windows *i* far enough away from the central window *i*^⋆^, we have the *α*_*i*_(*A*) = 0, and therefore $g_{i}^{\left(m,A\right)}=p$—*i.e*., the expectation of a neutral window. Based on these trends, windows far from the putatively selected target window are modeled as neutral, and windows close to the target window are heavily distorted due to the sweep. Moreover, because *α*_*i*_(*A*) tends to zero for windows far enough away for the central window, the model of neutrality is nested within our proposed sweep model. The schematic in Fig 1B illustrates the saltiLASSI framework of generating the spatially-distorted haplotype spectra.

Assume that in window i∈W, there is a *K*-truncated vector of counts

$$\begin{array}{c} x_{i}=\left(x_{i1},x_{i2},\ldots ,x_{iK}\right), \end{array}$$

which are the observed counts of the *K* most-frequent haplotypes, with *x*_*i*1_ ≥ *x*_*i*2_ ≥ ⋯ ≥ *x*_*iK*_ ≥ 0 and normalized such that $\sum_{k=1}^{K}x_{ik}=n_{i}$, where *n*_*i*_ is the total number of sampled haplotypes in window *i*. Following [33] and [13], we then compute the log composite likelihood ratios for null hypothesis of neutrality at target window *i*^⋆^ as

$$\begin{array}{c} \text{log}\;L_{0}(p,K;i^{⋆},{\left\{x_{i}\right\}}_{i\in W})=\sum_{i\in W}\sum_{k=1}^{K}x_{ik}\;\text{log}\left(p_{k}\right) \end{array}$$

and for the alternative hypothesis of *m* sweeping haplotypes at target window *i*^⋆^ as

$$\begin{array}{c} \text{log}\;L_{1}(p,K,\varepsilon ,m,A;i^{⋆},{\left\{x_{i},z_{i}\right\}}_{i\in W})=\sum_{i\in W}\sum_{k=1}^{K}x_{ik}\;\text{log}\;(g_{ik}^{\left(m,A\right)}). \end{array}$$

Using these log likelihoods, we follow [13] and construct a log likelihood ratio test statistic of a sweep at target window *i*^⋆^ as

$$\begin{array}{c} \Lambda =2[\text{log}\;L_{1}(p,K,\hat{\varepsilon },\hat{m},\hat{A};i^{⋆},{\left\{x_{i},z_{i}\right\}}_{i\in W})-\text{log}\;L_{0}(p,K;i^{⋆},{\left\{x_{i}\right\}}_{i\in W})], \end{array}$$

where

(m^,A^,ε^)=argmax(m,A,ε)logL1(p,K,ε,m,A;i⋆,{xi,zi}i∈W).

We note that this approach treats windows as independent in the null and alternative hypotheses, thus making it a composite likelihood method that ignores recombination.

### Computing the likelihood

To apply the saltiLASSI method, we compute Λ at each window in the genome, where each window is considered the target window *i*^⋆^ in turn, and the likelihood is maximized independently for each target window. That is, all parameters (*m*, *A*, and *ε*) are optimized at each target window *i*^⋆^, thereby permitting the footprint size *A* of the sweep to vary across the genome, adjusting for initial linkage disequilibrium and local recombination rates that could impact sweep signals. Similar to the way SweepFinder [17], SweepFinder2 [21], and LASSI [13] approach maximization, we optimize the likelihood via a grid search across *m* ∈ {1, 2, …, *K*}, *ε* ∈ [1/(100*K*), *U*], and *A* ∈ {*A*_min_, …, *A*_max_}. Here, *A*_min_ = −ln 0.99999/*d*_min_, representing a value of *A* with a slow decay with distance; *A*_max_ = −ln 0.00001/*d*_min_, representing a value of *A* with a fast decay with distance; and *d*_min_ is the smallest distance between any two windows genome-wide. We make 100 equally spaced (in log-space) steps between *A*_min_ and *A*_max_. Furthermore, in order to reduce computational burden, we pre-compute $q_{k}^{\left(m\right)}$ values across this grid for all windows.

### Power to detect sweeps

The power to detect sweeps will depend on a number of factors, including window size used to compute a statistic, whether phasing information for genotypes is used, the selection strength of the beneficial mutation *s*, the age of the sweep *t* (*i.e*., time at which the selected mutation became beneficial), the number of selected haplotypes *ν*, and the underlying demographic history. To explore the power of Λ, we evaluate its power to detect sweeps of varying strengths, softness, and ages. For sweep settings, we considered only simulations in which the beneficial mutation established by reaching a frequency of at least 0.1, but we did not condition on fixation. Under each setting, we interrogated its robustness to demographic history, both through idealized constant-size histories and histories with recent severe bottlenecks. Moreover we gauged whether Λ yields false sweep signals under settings of background selection. Furthermore, for each setting described, we investigated the power and robustness of using unphased multilocus genotypes as input to Λ instead of phased haplotypes. In addition, we evaluated the effect of sample size *n*, number of haplotypes *K* to truncate the HFS, and recombination rate variation on the power of Λ to detect sweeps. Finally, we compared Λ to competing contemporary methods that use the same type of input data, using the *T* statistic of [13] for phased and unphased input data, and also considered the H12 [8], *nS*_*L*_ [7], and iHS [5] statistics for phased data and the G123 statistic [10] for unphased data. The simulation protocol for all settings is described in the *Methods* section.

**Fig 2:**
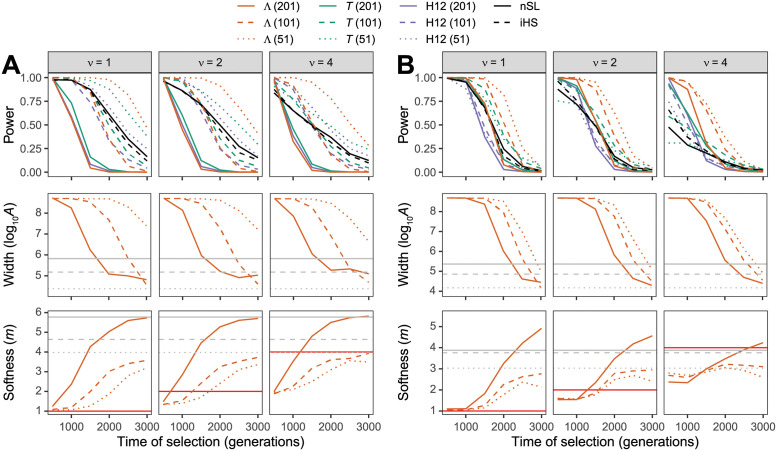
Performance of detecting and characterizing sweeps. Performance for applications of Λ, *T*, and H12 with windows of size 51, 101, and 201 SNPs, as well *nS*_*L*_ and iHS under simulations of (A) a constant-size demographic history or (B) the human central European (CEU) demographic history of [34]. Results are based on a sample of *n* = 50 diploid individuals and the haplotype frequency spectra for the Λ and *T* statistics truncated at *K* = 10 haplotypes. (Top row) Power at a 1% false positive rate as a function of selection start time. (Middle row) Estimated sweep width illustrated by mean estimated genomic size influenced by the sweep (${\text{log}}_{10}\;\hat{A}$) as a function of selection start time. Gray solid, dashed, and dotted horizontal lines are the corresponding mean ${\text{log}}_{10}\;\hat{A}$ values for Λ applied to neutral simulations. (Bottom row) Estimated sweep softness illustrated by mean estimated number of sweeping haplotypes ($\hat{m}$) as a function of selection start time. Gray solid, dashed, and dotted horizontal lines are the corresponding mean $\hat{m}$ values for Λ applied to neutral simulations, and the red solid horizontal lines correspond to the number of sweeping haplotypes *ν* ∈ {1, 2, 4} assumed in sweep simulations. Sweep scenarios consist of hard (*ν* = 1) and soft (*ν* ∈ {2, 4}) sweeps with per-generation selection coefficient of *s* = 0.1 that started at *t* ∈ {500, 1000, 1500, 2000, 2500, 3000} generations prior to sampling. Results expanded across wider range of simulation settings can be found in S1–S3 and S7–S9 Figs as well as results for application to unphased multilocus genotype data in S4–S6 and S10–S12 Figs.

To begin, we compare the performance of Λ to *T*, H12, *nS*_*L*_, and iHS under a constant-size demographic history with diploid effective size of *N* = 10^4^ diploid individuals. The Λ, *T*, and H12 statistics were computed for different window sizes, consisting of 51, 101, or 201 SNPs per window. Fig 2A and S1 Fig show that across sweeps of varying degrees of softness (beneficial mutation on *ν* ∈ {1, 2, 4, 8, 16} distinct haplotypes) and for sweeps of varying per-site per-generation strengths of *s* ∈ {0.01, 0.1}, the method with highest power regardless of time of selection (*t* ∈ {500, 100, 1500, 2000, 2500, 3000} generations prior to sampling) is Λ, thereby outperforming the competing methods. Interestingly, Λ applied to 51 SNP windows has generally higher power than with 101 and 201 SNP windows. Furthermore, smaller window sizes enable Λ to achieve high power even for old sweeps—with this elevated power often substantially higher than the closest competing method. This result recapitulates a finding of [13], where they observed that if the spatial distribution of the *T* statistic was used within a machine learning framework, computing the *T* statistic in a greater number of small windows yielded higher power for ancient sweeps than when a smaller number of large windows was used. This is an intriguing result, because smaller windows have poorer estimates of the distortion of the HFS, yet it appears that for detecting ancient sweeps what matters is capturing the overall spatial trend of the distortion of the HFS. That is, when using too large of windows, Λ is averaging the HFS across too large of a region, which has likely been broken up over time due to recombination for ancient sweeps. Instead, smaller windows focus on genomic segments with less shuffling of haplotype variation due to recombination events, such that distortions in the HFS are due to the effect of a sweep at a nearby selected site.

S1 Fig also highlights a key distinction among sweeps of different strengths. Specifically, regardless of method considered, each achieves its highest power when sweeps of strength *s* = 0.1 are recent, whereas for sweeps of strength *s* = 0.01, highest power for each method is shifted farther in the past toward more ancient sweep. This pattern was also found previously for H12 [10] and *T* [13]. The likely reason for this result is that sweeps of strength *s* = 0.01 require more time for the beneficial allele to reach high frequency and leave a conspicuous genomic footprint, with this greater time to reach high frequency associated with increased chance that recombination and mutation act to break up high-frequency haplotypes. In contrast, sweeps of strength *s* = 0.1 create an immediate selection signature to appear in the genome due to the rapid rise in frequency of a beneficial mutation, but traces of this sweep pattern erode over time due to recombination, mutation, and drift. However, regardless, the Λ statistic paired with a small window size yields uniformly better or comparable sweep detection ability than the other approaches we examined. We also found that all methods performed poorly when selection strength was *s* = 0.001.

During a scan with Λ, the composite likelihood ratio is optimized over the number of high frequency (sweeping) haplotypes *m* and the footprint size of the sweep *A*, leading to respective estimates $\hat{m}$ and $\hat{A}$. Therefore, at a genomic location with evidence for a sweep (high Λ value), we may better understand properties of the putative sweep by evaluating its softness through $\hat{m}$ and its strength or age through $\hat{A}$. S2 Fig shows that for sweeps of strength *s* = 0.01, the estimated number of sweeping haplotypes $\hat{m}$ is considerably different from the actual number of initially-selected haplotypes *ν*, regardless of window size used or age of the sweep. In contrast, Fig 2A and S2 Fig reveal that for hard sweeps (*ν* = 1) of strength *s* = 0.1, the estimate of the number of sweeping haplotypes when using 51 SNP windows is often consistent with hard sweeps ($\hat{m}=1$) provided that the sweep is recent enough (within the last 500 generations). Similarly, under these same settings but with soft sweeps of *ν* ∈ {2, 4, 8, 16} selected haplotypes (Fig 2A and S2 Fig), the estimated number of sweeping haplotypes tends to be underestimated ($\hat{m}<\nu$) but is still consistent with a soft sweep ($\hat{m}>1$). Therefore, provided that a sweep is recent enough, when using 51 SNP windows the value of the estimated number of sweeping haplotypes can be used to lend evidence of a hard ($\hat{m}=1$) or a soft ($\hat{m}>1$) sweep.

Similarly, the other parameter estimate $\hat{A}$ may also help characterize identified sweeps. Specifically, Fig 2A and S3 Fig show that the footprint size of the sweep (measured as ${\text{log}}_{10}\;\hat{A}$) is substantially elevated compared to expectation for neutral simulations for sweep times at which there is high power to detect sweeps (Fig 2A and S1 Fig). Interestingly, the shape of the curves relating the mean sweep footprint size over time mirror the power of the Λ statistic with corresponding window size as a function of sweep initiation time (*t*), sweep softness (*ν*), and sweep strength (*s*). These results suggest that the estimate of the sweep footprint size (${\text{log}}_{10}\;\hat{A}$) can be used to learn about the age or strength of a candidate sweep (the signatures of which appear to be confounded between the two parameters). Coupled with an estimate of the sweep softness ($\hat{m}$), our saltiLASSI framework provides a means to not only detect sweeps with high power, but to also learn the underlying parameters that may have shaped the adaptive evolution of candidate sweep regions.

Obtaining phased haplotypes for input to Λ represents an error-prone step that, without sufficient reference panels or high-enough quality genotypes, may make identification of sweeps difficult or potentially impossible for a number of diverse study systems. It is therefore beneficial if the favorable performance of Λ transfers to datasets that have not been phased. Similar to prior studies (e.g., [10, 13, 29, 32], we sought to evaluate the power of Λ when applied to unphased multilocus genotype data, and to compare its performance with the *T* statistic and G123 (analogue of H12 for use with unphased data) [10], both of which are also applied to unphased multilocus genotypes. S4 Fig shows that Λ maintains high power to detect sweeps of differing ages, strengths, and softness. Consistent with the results on haplotype data (Fig 2A and S1 Fig), Λ generally displays higher power than, or comparable power to, *T* and G123, with the best performance deriving from Λ with a small window size of 51 SNPs, and with substantially higher power for old sweeps compared to other approaches. An exception is that for recent (*t* ≤ 1000 generations) and highly soft (*ν* = 16) sweeps, using a window size of 101 SNPs for Λ had substantially higher power than using the smaller 51 SNP window. Moreover, for highly soft (*ν* = 16) and ancient (*t* ≥ 2000) sweeps with strength *s* = 0.1, the power of Λ is much lower with unphased multilocus genotypes compared to phased haplotypes (compare S1 and S4 Figs). Interpretation of $\hat{m}$ is more difficult for multilocus genotypes compared to haplotypes. However, consistent with the results for haplotypes (S2 and S5 Figs) shows that when using 51 SNP windows, Λ tends to estimate a small number of sweeping multilocus genotypes (smaller $\hat{m}$) for harder sweeps (smaller *ν*) than for softer sweeps (larger *ν*).

While adaptive processes generally affect variation locally in the genome, neutral processes such as demographic history influence overall levels of genome diversity. Specifically, it is common to consider that demographic processes impact the mean value of genetic diversity, and numerous likelihood approaches for detecting sweeps [13, 16–24] and other forms of natural selection [33, 35, 36] have been created to specifically account for this average effect of demographic history on genome diversity. However, demographic processes, such as recent severe bottlenecks, not only alter mean diversity but also influence higher-order moments of diversity, potentially making it insufficient to account solely for the mean effect of diversity [37–39]. Given that Λ does not account for higher moments than the mean effect of demographic history on the HFS, we sought to evaluate its properties under recent strong bottlenecks—a setting that has proven challenging for other sweep statistics in the past.

The Λ statistic generally exhibits superior power to *T*, H12, *nS*_*L*_, and iHS when applied to haplotype data (Fig 2B and S7 Fig) or to *T* and G123 when applied to unphased multilocus genotype data (S10 Fig). Moreover, the general trends in method power as a function sweep strength, softness, and age observed for the constant-size history (Fig 2A, S1 and S4 Figs) hold for this complex demographic setting (Fig 2B, S7 and S10 Figs), with the caveat that, as expected, power for all methods is generally lower under the bottleneck compared to the constant-size history. A clear difference between these two demography settings is that, whereas Λ had exhibited uniformly superior or comparable power with smaller 51 SNP windows compared to larger 101 or 201 SNP windows (Fig 2A and S1 Fig), under the bottleneck model the best window size depends on age of the sweep (Fig 2B and S7 Fig). In particular, recent sweeps often had highest power with 201 SNP windows, sweeps of intermediate age with 101 SNPs, and ancient sweeps with 51 SNPs. Therefore, under complex demographic histories, choice of window size for Λ is more nuanced than with constant-size histories. This result is consistent with those of [13] who demonstrated that, when accounting for the spatial distribution of the *T* statistic in a machine learning framework (referred to as *T*-*Trendsetter*), power to detect recent sweeps is higher for larger windows and power to detect ancient sweeps is higher for smaller windows under the bottleneck history considered here.

In addition to demographic history, a pervasive force acting to reduce variation across the genome is background selection [40–43], which is the loss of genetic diversity at neutral sites due to negative selection at nearby loci [44–46]. Background selection has been demonstrated to alter the neutral SFS [44, 47–49], and masquerade as false signals of positive selection [19, 44–47, 50–54]. However, because this process does not generally lead to haplotypic variation consistent with sweeps [55–57], like prior studies developing haplotype approaches for detecting sweeps [10, 13] we sought to evaluate the robustness of Λ to background selection. We find that under both simple and complex demographic histories, using either phased haplotype or unphased multilocus genotype data, all methods considered here demonstrate robustness to background selection by not falsely attributing genomic regions evolving under background selection as sweeps (S19 Fig).

Throughout our experiments, we have considered a per-site per-generation recombination rate of *r* = 10^−8^ for each simulation replicate. However, recombination rate is known to vary across the genome [58], and it is therefore important to evaluate the performance of Λ compared to other methods when recombination rate varies across genomic regions. To evaluate the effect of recombination rate variation on method performance, we drew per-site per-generation recombination rate from an exponential distribution with mean 10^−8^ (see Methods) for reach replicate neutral and sweep simulation under the bottleneck demographic history [34]. S13 and S16 Figs indicate that the Λ statistic generally has greater power than *T*, H12 (or G123), *nS*_*L*_, and iHS under phased haplotypes and unphased multilocus genotypes settings. These results further highlight the robustness of the Λ statistic to realistic genomic characteristics often encountered in empirical studies.

**Fig 3:**
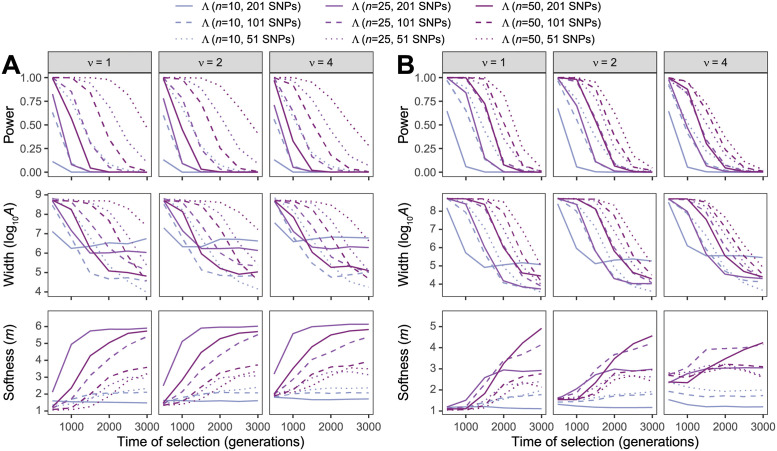
Performance of detecting and characterizing sweeps. Performance for applications of Λ with windows of size 51, 101, and 201 SNPs under simulations of (A) a constant-size demographic history or (B) the human central European (CEU) demographic history of [34] and sample size of *n* ∈ {10, 25, 50} diploid individuals. Results are based on the haplotype frequency spectra for the Λ statistic truncated at *K* = 10 haplotypes. (Top row) Power at a 1% false positive rate as a function of selection start time. (Middle row) Estimated sweep width illustrated by mean estimated genomic size influenced by the sweep (${\text{log}}_{10}\;\hat{A}$) as a function of selection start time. (Bottom row) Estimated sweep softness illustrated by mean estimated number of sweeping haplotypes ($\hat{m}$) as a function of selection start time. Sweep scenarios consist of hard (*ν* = 1) and soft (*ν* ∈ {2, 4}) sweeps with per-generation selection coefficient of *s* = 0.1 that started at *t* ∈ {500, 1000, 1500, 2000, 2500, 3000} generations prior to sampling. Results expanded across wider range of simulation settings can be found in S20–S22 and S26–S28 Figs as well as results for application to unphased multilocus genotype data in S23–S25 and S29–S31 Figs.

**Fig 4:**
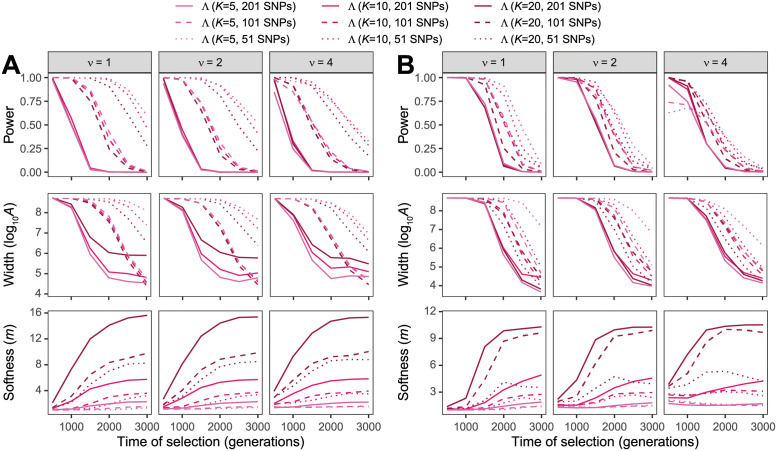
Performance of detecting and characterizing sweeps. Performance for applications of Λ with windows of size 51, 101, and 201 SNPs under simulations of (A) a constant-size demographic history or (B) the human central European (CEU) demographic history of [34] and the haplotype frequency spectra for the Λ statistic truncated at *K* ∈ {5, 10, 20} haplotypes. Results are based on a sample of *n* = 50 diploid individuals. (Top row) Power at a 1% false positive rate as a function of selection start time. (Middle row) Estimated sweep width illustrated by mean estimated genomic size influenced by the sweep (${\text{log}}_{10}\;\hat{A}$) as a function of selection start time. (Bottom row) Estimated sweep softness illustrated by mean estimated number of sweeping haplotypes ($\hat{m}$) as a function of selection start time. Sweep scenarios consist of hard (*ν* = 1) and soft (*ν* ∈ {2, 4}) sweeps with per-generation selection coefficient of *s* = 0.1 that started at *t* ∈ {500, 1000, 1500, 2000, 2500, 3000} generations prior to sampling. Results expanded across wider range of simulation settings can be found in S32–S34 and S38–S40 Figs as well as results for application to unphased multilocus genotype data in S35–S37 and S41–S43 Figs.

Finally, the number *n* of sampled individuals as well the number *K* of haplotypes used to truncate the HFS should affect the resolution at which we can model the distortion of the HFS due to a sweep, and thus would likely result in alterations of power of Λ to detect sweeps. As expected, Fig 3 shows that increasing sample size generally increases power of Λ to detect sweeps, with highest power typically obtained with the largest *n* and smallest window size combination (*i.e*., *n* = 50 with 51-SNP windows) and the lowest power with the smallest *n* and largest window size combination (*i.e*., *n* = 10 with 201-SNP windows). Moreover, as sample size increases, Λ is better able to detect sweeps of older age, and for extremely small samples (*i.e*., *n* = 10), the estimates $\hat{m}$ of the number *ν* of sweeping haplotypes are poor. In contrast to changing sample size *n*, changing the number of haplotypes *K* to truncate the HFS does not have a substantial effect on the power of Λ to detect sweeps (Fig 4, with the power curves for a specific window size mostly the same across *K* ∈ {5, 10, 20}. This result mirrors that in S5 Fig of [13] for the *T* statistic, whereby changing *K* had little effect on method power. Instead, choice of *K* seems to more strongly influence the estimates $\hat{m}$ of the number *ν* of sweeping haplotypes, with larger values of *K* permitting a wider range of estimates of *m*. This result mimics those observed for the *T* statistic by [13], in that the choice of *K* has a larger effect on the resolution to classify sweeps as hard or soft than it did on the ability to detect sweeps.

### Application to empirical data

#### Humans from the 1000 Genomes Project

The 1000 Genomes Project Phase 3 [31] published the whole genomes of 2504 humans across 26 populations around the world. To illustrate the use of the saltiLASSI framework in a context where the populations of interest have well-studied demographic histories, we calculate Λ in two populations: a European population (CEU; *n* = 99) and an African population (*n* = 108). Furthermore, as patterns of recent selection have been extensively studied in these populations, the results will allow us to confirm that the method returns sensible results.

**Fig 5:**
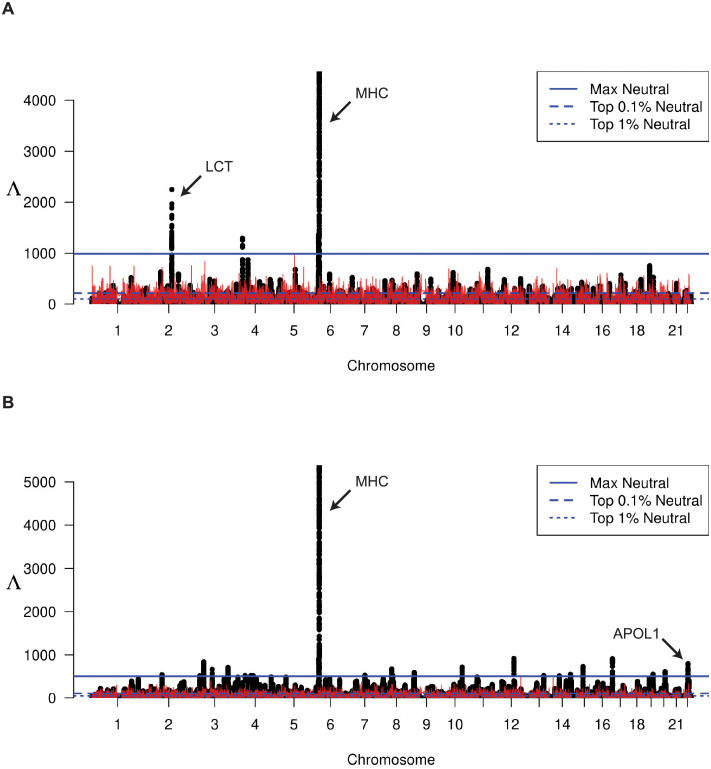
Manhattan plot of Λ-statistics. For the (A) CEU and (B) YRI populations from the 1000 Genomes Project. Each point represents a single 201-SNP window along the genome. Horizontal lines represent the top 1%, top 0.1%, and maximum observed Λ statistic across all windows in demography-matched neutral simulations. Red line indicates the maximum observed Λ among 100 replicate simulations at that location in the genome.

We plot the genome-wide Λ statistics for the CEU population in Fig 5A and the YRI population in Fig 5B. We find several conspicuous peaks of notably large Λ values, which indicates strong support for a highly distorted HFS in these regions compared to the genome-wide mean HFS. We plot the local maximum Λ observed across simulations as a red line, the over-all maximum score (horizontal solid blue line), over-all top-0.1% (horizontal dashed blue line), and over-all top-1% (horizontal dotted blue line); see Methods for details.

As this statistic is a composite likelihood ratio test that ignores recombination, we expected that Λ values may be negatively correlated with recombination rate. And, indeed, we find that the max Λ observed in a window across all replicates tends to be larger for low-recombination regions S46 Fig. With this in mind, we chose a conservative threshold for determining significance by only calling regions as under selection when the observed Λ is greater than the over-all genome-wide maximum observed Λ from neutral simulations. Taking this approach, we identify several regions in both populations with scores consistently above this threshold, including five regions in the CEU population (Table 1) and 29 in the YRI population (Table 2). Among these regions, we find several well-studied genes that are known to have been under selection in these populations. These include the lactase gene (*LCT* [9, 59–61]), the major histocompatibility complex (MHC [9, 61, 62]), and the apolipoprotein L1 (*APOL1* [63]). We next conduct a gene ontology over-representation test for molecular function using PANTHER16 [64] for each population separately. We find that each population’s putatively selected genes are generally representing similar molecular functions (S2 and S3 Tables), including MHC class II receptor activity, MHC class II protein complex binding, and peptide antigen binding, further underscoring the evidence for immune system adaptation in human populations around the world [9, 61, 62, 65].

**Table 1:** Regions of extreme Λ values in the CEU population and the genes contained therein. $\hat{m}$ is the inferred number of sweeping haplotypes, and ${\text{log}}_{10}\left(\hat{A}\right)$ is the estimated sweep width.

Chr	Start (bp)	Stop (bp)	$\hat{m}$	${\text{log}}_{10}\left(\hat{A}\right)$	Max Λ	Genes
2	135,517,106	136,318,189	1	7.817	1889.370	*ACMSD*, *MIR5590*, *CCNT2-AS1*, *CCNT2*, *MAP3K19*, *RAB3GAP1*, *ZRANB3*, *R3HDM1*
2	136,524,766	136,816,336	1	7.817	2250.050	*UBXN4*, *LCT*, *LOC100507600*, *MCM6*, *DARS*, *DARS-AS1*
4	34,296,435	34,400,578	1	8.252	1296.390	–
6	29,782,470	29,996,854	9	7.817	1366.550	*HLA-G*, *HLA-H*, *HCG4B*, *HLA-A*, *HCG9*, *ZNRD1-AS1*, *HLA-J*, *HCG8*
6	32,384,933	32,723,916	7	7.817	4565.340	*HLA-DRA*, *HLA-DRB5*, *HLA-DRB6*, *HLA-DRB1*, *HLA-DQA1*, *HLA-DQB1*, *HLA-DQB1-AS1*, *HLA-DQA2*, *MIR3135B*, *HLA-DQB2*

**Table 2:** Regions of extreme Λ values in the YRI population and the genes contained therein. $\hat{m}$ is the inferred number of sweeping haplotypes, and ${\text{log}}_{10}\left(\hat{A}\right)$ is the estimated sweep width.

Chr	Start (bp)	Stop (bp)	$\hat{m}$	${\text{log}}_{10}\left(\hat{A}\right)$	Max Λ	Genes
2	89,247,854	89,309,423	8	7.817	542.403	–
3	27,184,951	27,213,780	7	8.252	518.153	*NEK10*
3	46,080,758	46,384,651	6	8.252	839.195	*CCR1*, *CCR3*
3	87,273,225	87,328,290	6	8.252	665.802	*MIR4795*, *CHMP2B*, *POU1F1*
3	162,536,420	162,681,511	6	8.252	711.747	–
3	163,811,348	163,832,931	7	8.252	515.029	–
4	46,828,990	46,881,572	8	8.252	521.042	*COX7B2*
4	74,441,768	74,503,196	7	8.252	520.893	*RASSF6*
4	86,144,358	86,185,751	9	8.252	517.750	–
6	31,193,373	31,387,666	11	7.817	918.650	*HLA-C*, *HLA-B*, *MIR6891*, *MICA*
6	32,370,521	32,750,144	8	7.817	5385.050	*BTNL2*, *HLA-DRA*, *HLA-DRB5*, *HLA-DRB6*, *HLA-DRB1*, *HLA-DQA1*, *HLA-DQB1*, *HLA-DQB1-AS1*, *HLA-DQA2*, *MIR3135B*, *HLA-DQB2*
6	32,973,878	33,206,733	8	7.817	1196.890	*HLA-DOA*, *HLA-DPA1*, *HLA-DPB1*, *HLA-DPB2*, *COL11A2*, *RXRB*, *SLC39A7*, *HSD17B8*, *MIR219A1*, *RING1*
7	80,311,522	80,335,123	7	8.252	525.732	–
7	80,335,124	80,390,838	7	8.252	527.827	*SEMA3C*
8	50,054,576	50,219,674	6	8.252	672.218	–
8	54,726,028	54,770,306	7	8.252	526.734	*ATP6V1H*, *RGS20*
9	11,767,302	11,832,748	8	8.252	594.456	–
10	102,156,400	102,295,419	5	8.252	717.760	*WNT8B*, *SEC31B*, *NDUFB8*
12	79,566,706	79,800,343	6	8.252	917.585	*SYT1*
13	89,191,303	89,233,281	8	8.252	521.733	*LINC00433*
14	48,798,901	48,833,362	8	7.817	504.040	–
14	48,833,363	48,853,450	7	7.817	515.715	–
14	102,173,879	102,216,165	7	7.817	551.532	*LINC00239*
15	55,137,170	55,292,403	7	8.252	730.824	–
17	3,515,275	3,672,429	6	8.252	911.490	*SHPK*, *CTNS*, *TAX1BP3*, *P2RX5-TAX1BP3*, *EMC6*, *P2RX5*, *ITGAE*, *GSG2*
19	38,859,266	38,939,066	7	8.252	555.198	*CATSPERG*, *PSMD8*, *GGN*, *SPRED3*, *FAM98C*, *RASGRP4*, *RYR1*
19	39,176,160	39,201,469	7	8.252	504.612	*ACTN4*
20	37,392,189	37,490,122	5	8.686	613.034	*ACTR5*, *PPP1R16B*
22	36,567,890	36,756,255	6	8.252	796.478	*APOL4*, *APOL2*, *APOL1*, *MYH9*, *MIR6819*

**Fig 6:**
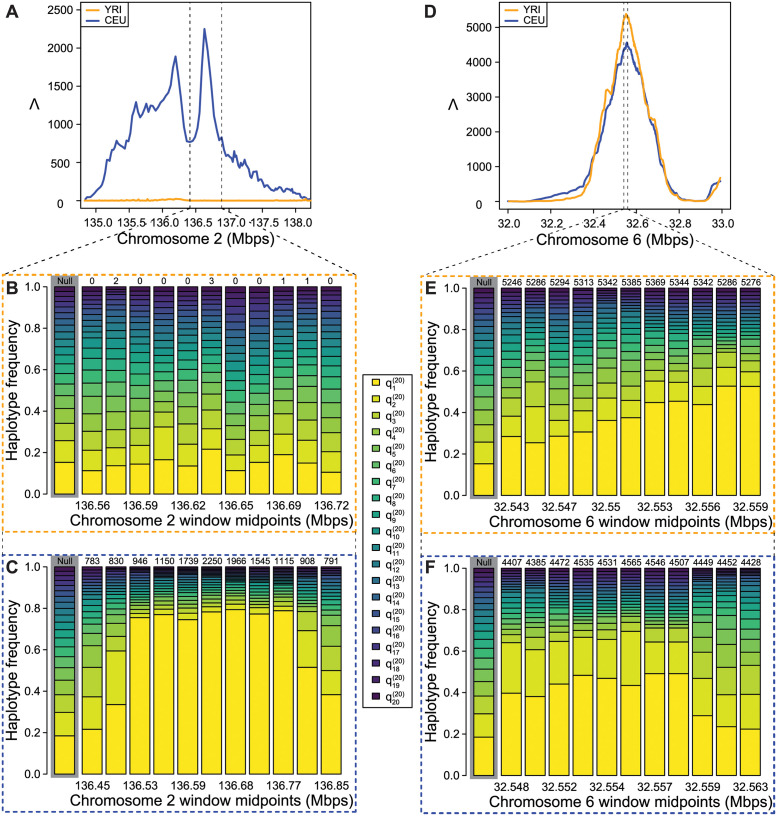
Detailed illustration of Λ statistics and haplotype frequency spectra in CEU and YRI. (A) Λ plotted in the *LCT* region, vertical dotted lines indicate zoomed region shown in (B) and (C). (B) YRI empirical HFS for 11 windows in the *LCT* region. (C) CEU empirical HFS for 11 windows in the *LCT* region. (D) Λ plotted in the MHC region, vertical dotted lines indicate zoomed region shown in (E) and (F). (E) YRI empirical HFS for 11 windows in the MHC region. (F) CEU empirical HFS for 11 windows in the MHC region. In (B), (C), (E), and (F), numbers above HFS are Λ values for the window rounded to the nearest whole number, and the genome-wide average HFS is highlighted in grey. $q_{i}^{20}$ is the frequency of the *i*th most common haplotype truncated to *K* = 20.

We next explore two peaks in detail, the *LCT* and MHC loci (Fig 6), to illustrate the spatial structure of the HFS in these regions of strong signal in one (*LCT*) or both (MHC) populations. The *LCT* locus has been previously identified as under selection in some northern European populations and eastern African populations [59]. As the CEU population has largely northern European ancestry and the YRI population is from western Africa, we expect to find a peak near *LCT* in CEU but not in YRI. Indeed, this is what we see in Fig 6A, which plots Λ statistics in the vicinity of the *LCT* locus on Chromosome 2. Furthermore, we examine the truncated HFS among eleven windows spanning *LCT* in both YRI (Fig 6B) and CEU (Fig 6C). We see in Fig 6B that YRI has haplotype frequencies similar to the genome-wide mean (plotted and highlighted on the left), whereas Fig 6C shows that the CEU population is dominated largely by a single haplotype near 80% frequency. Indeed, the saltiLASSI method also infers a $\hat{m}=1$ in this region (Table 1), indicating a single sweeping haplotype (i.e., a hard sweep). Furthermore, we can see the HFS in this region trending toward the genome-wide mean as the windows move farther from the sweep’s focal point, illustrating the pattern that the saltiLASSI method was designed to capture.

Fig 6D–6F illustrate the Λ statistics and HFS patterns in the vicinity of the MHC locus. This locus contains a large cluster of immune system genes, and selection at this locus is distinguished from *LCT* in that high diversity is preferred in order for the body to be able to mount a robust response to unknown pathogen exposure. As expected, both populations have extreme Λ values (Fig 6D) and a greatly distorted HFS in this region (Fig 6E and 6F). However, we note that the HFS is clearly distorted in favor of multiple haplotypes, in contrast to *LCT*, which we expect at a locus that favors diversity. Indeed, the saltiLASSI method infers $\hat{m}$ to be between seven and nine in the CEU population and between eight and 11 for YRI (variance due to multiple regions within the MHC being separately identified; Tables 1 and 2).

We repeated our analyses of these two populations and two loci using the unphased multilocus-genotype approach (S44 and S45 Figs and S5 and S6 Tables), and we find good concordance with the phased haplotype approach.

Finally, we re-compute Λ (phased) in these two populations’ empirical data and all replicates of simulated demography-matched whole-genome data using two distance measures other than physical distance (number of windows and centiMorgans) and find high correlation between Λ values calculated with these alternative distance measures and physical distance (S4 Table).

#### Rats from New York City

[32] published a whole-genome dataset of brown rat samples (*n* = 29) from across the island of Manhattan, New York City, USA to study adaptation to urban environment. In this study, they note that haplotype phase is unknown and that the demographic history for brown rats was not well-calibrated in this population. As such, they chose to use the G123 [10] and other statistics, which used multilocus genotypes combined with a gene-based outlier approach to identify putative targets of selection. Here, we re-analyze this data using the saltiLASSI framework to illustrate its use in the context of unphased data and a poorly understood demographic history that requires an outlier approach.

**Fig 7:**
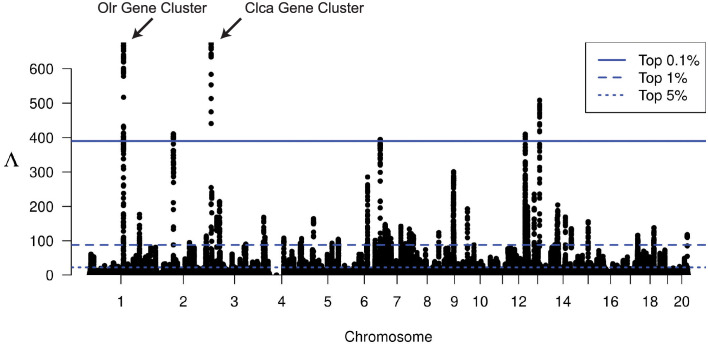
Manhattan plot of Λ-statistics for the New York City rat population. Each point represents a single 201-SNP window along the genome. Horizontal lines represent the top 5%, top 1%, and top 0.1% observed Λ statistic across all windows in the genome.

We plot the genome-wide Λ statistics for the NYC rats in Fig 7, along with blue horizontal lines indicating the top 0.1% (solid), top 1% (dashed), and top 5% (dotted) empirically observed Λ values genome-wide. We identify putatively selected regions as windows with a Λ greater than the top 1% empirical threshold (see Methods), with consecutive windows satisfying this condition concatenated together. These regions are then annotated with known genes (RN5 genome build) and presented in S7 and S8 Tables.

We note that the two strongest signals in the genome are on chromosomes 1 and 2 (S7 Table). The region on chromosome 1 contains a cluster of olfactory receptor genes (*Olr23*, *Olr24*, *Olr25*, *Olr27*, *Olr29*, *Olr30*, *Olr32*, and *Olr34*), and the region on chromosome 2 contains a cluster of calcium-activated chloride channel genes (*Clca2*, *Clca4l*, *Clca4*, *Clca1*, and *Clca5*). Notably, calcium-activated chloride channel genes are expressed in the olfactory nerve layer of mouse brains [66]. If these calcium-activated chloride channel genes are similarly expressed in rats, then these two strong selection signals suggest that this urban rat population may be experiencing selection pressures associated with olfactory perception.

Taking the collection of annotated genes present in S7 Table, we conduct a gene ontology over-representation test based on molecular function category using PANTHER16 [64] with results presented in Table 3. We find that Intracellular Calcium Activated Chloride Channel Activity, Peptidase Activity, and Odorant Binding are statistically over-represented molecular functions among this set of putatively selected genes.

**Table 3:** Gene ontology enrichment analysis of regions with extreme Λ values in the New York City rat population.

GO molecular function	Fold Enrichment	Raw P-value	FDR
Intracellular calcium activated chloride channel activity	>100	5.16 × 10^−12^	1.23 × 10^−8^
Intracellular chloride channel activity	>100	5.16 × 10^−12^	2.46 × 10^−8^
Chloride channel activity	45.49	5.81 × 10^−9^	6.93 × 10^−6^
Anion channel activity	39.07	1.37 × 10^−8^	1.31 × 10^−5^
Inorganic anion transmembrane transporter activity	24.06	2.14 × 10^−7^	1.70 × 10^−4^
Inorganic molecular entity transmembrane transporter activity	6.39	2.96 × 10^−5^	9.42 × 10^−3^
Anion transmembrane transporter activity	11.30	1.51 × 10^−5^	6.53 × 10^−3^
Ion transmembrane transporter activity	5.47	8.71 × 10^−5^	2.60 × 10^−2^
Ion channel activity	9.07	1.10 × 10^−5^	5.27 × 10^−3^
Channel activity	8.12	2.23 × 10^−5^	7.60 × 10^−3^
Passive transmembrane transporter activity	8.12	2.23 × 10^−5^	8.19 × 10^−3^
Ion gated channel activity	69.19	5.57 × 10^−10^	8.88 × 10^−7^
Gated channel activity	11.60	2.27 × 10^−6^	1.21 × 10^−3^
Metallopeptidase activity	16.86	1.60 × 10^−6^	9.56 × 10^−4^
Peptidase activity	6.92	1.68 × 10^−5^	6.70 × 10^−3^
Odorant binding	10.06	1.12 × 10^−6^	7.62 × 10^−4^

## Discussion

In this study, we developed a new likelihood ratio test statistic Λ that examines the spatial distribution of the HFS for evidence of sweeps. We demonstrated that this statistic has high power to detect both hard and soft sweeps, with performance substantially better than competing haplotype-based approaches for the same task. Moreover, while optimizing the model parameters of Λ we obtain estimates of sweep softness *m* and footprint size *A*, which is correlated with age and strength of the sweep. These additional parameters have the potential to further characterize well-supported sweep signals from large Λ values.

In addition to lending exceptional performance on simulated data, application of Λ to whole-genome variant calls from central European and sub-Saharan African individuals recapitulated the well-established signal at the *LCT* gene in Europeans due to lactase persistence [67], as well as sweep footprints at the MHC locus in both populations related to immunity, which have previously been detected with other sweep statistics [13, 68, 69]. Though not novel findings, the clear (Fig 6) and strong (Fig 5) signals at these two loci serve as positive controls to highlight the efficacy of Λ. Furthermore, these findings were similarly recapitulated with unphased multilocus genotype data (S44 and S45 Figs), lending support for the utility of Λ when applied to study systems for which obtaining phased haplotypes data is challenging.

Though our identification of the MHC locus in both human empirical scans as a sweep is not novel, it is important to address that the MHC locus comes with a number of technical challenges when assessing genetic variation. Specifically, the MHC locus is known to harbor extensive structural variation, which makes it difficult to assemble [70] and may lead to downstream errors in variant and genotype calling and in haplotype phasing. Indeed, such difficult to assemble regions may lead to enrichment in heterozygous sites, where in the extreme the majority of individuals are heterozygous. Contiguous SNPs in which individuals have heterozygous genotypes may manifest as a single high-frequency unphased multilocous genotype that stems from two distinct and divergent high-frequency haplotypes. Because Λ only considers the frequency of haplotypes and multilocus genotypes, it may lend support for sweeps in regions where genetic variation is difficult to assay. As with any other sweep detection approach, we recommend that care be taken when pre- and post-processing genomic datasets to attempt to circumvent these issues whenever possible, such as filtering regions with poor mappability, as we have done in this study (see Methods).

As the human populations have a well-characterized demographic history, we were able to perform demography-matched neutral simulations to aid in identifying regions of the genome likely affected by selection. When analyzing the New York City brown rat dataset, we had to take an outlier approach as the brown rat demographic history was previously noted to be mis-calibrated for this population [32]. However, our outlier approach notably identified two strong signals of selection among clusters of genes related to olfactory perception. As rats depend heavily on scents for communication and behavior choices [71–73], it is reasonable to think that a harsh, noisy, urban environment may present selection pressure on this biological system.

A key parameter that must be chosen when applying Λ is the number of SNPs per window. Specifically, we found that larger windows had greatest power for more recent sweeps, and smaller windows for more ancient sweeps (Fig 2, S1 and S7 Figs), mirroring the window size results observed in S8 and S9 Figs of [13] for the spatial distribution of the *T* statistic using a different modeling approach. Therefore, choice of window size may be informed by the time frame of selective events that is being investigated. As highlighted in Fig 2B and S7 Fig, the Λ statistic computed within windows of 201 SNPs had highest power of all other tested window sizes within the past 1500 generations under the central European demographic history. Because selective events within this time frame are consistent with adaptive events in recent evolution of modern humans [74–76], we selected this size so that we could recapitulate expected well-established sweeps—*e.g*., Figs 5 and 6 highlighting the sweep signal at *LCT*. In addition to using simulation results to aid in selecting appropriate window sizes, an alternate method such as choosing sizes based on the expected decay of linkage disequilibrium in the genome has been demonstrated to also work well in practice (e.g., [8, 13]).

We note that this approach is a composite likelihood statistic, and as such it treats windows as independent, ignoring the effects of recombination. This means that Λ values are likely to be larger in low-recombination regions (S46 Fig), and extreme scores found in such regions should be treated with extra scrutiny. However, even in such regions, we have shown that one can employ a simulation based approach to evaluate the uncertainty in the estimated Λ values (Fig 5 and S44 Fig)—albeit such an approach can be computationally intensive and would require accurate demographic model and recombination map estimates. An alternative solution to evaluate the uncertainty in Λ while also accounting for recombination would be to perform a block resampling locally in the genome [77]. Such an approach would prove valuable for study systems without accurate estimates of demographic models and recombination maps, and would provide an alternative uncertainty metric even for organisms such as humans for which simulations can be employed to evaluate uncertainty.

The *T* statistic of [13] presented the first likelihood approach that evaluated distortions in the HFS to detect selective sweeps, importantly because neutrality and soft sweeps leave similar signatures in the SFS but different within the HFS [78]. As demonstrated by [13], using the spatial distribution of the *T* statistic within a machine learning framework enhanced its detection ability, specifically for ancient sweeps. However, machine learning frameworks require extensive simulations to train (e.g., [25, 27, 28]), and these simulations must be based on a set of critical assumptions, such as demographic, mutation rate, and recombination rate parameters. Yet, accurate inferences of these parameters is not always possible, or can be highly error prone, and prior studies have found that these machine learning methods can make highly incorrect predictions if the distribution of training data is different from that of the test or empirical data [25, 30]. Furthermore, generation of these training datasets and training the models on them often requires substantial computational time and resources. Instead, our Λ statistic is the first likelihood method to model the spatial distribution of the HFS, providing the power of modeling the spatial distribution of *T* afforded by current machine learning frameworks (*e.g*., compare S1 and S7 Figs with S8 and S9 Figs of [13]). This power comes without having to simulate over a broad range of parameters to train a model, thus saving computational resources, and with predictions not hinging on accurate estimates of genetic and evolutionary model parameters to generate training sets. However, this high power of the Λ statistic to detect candidate sweep regions without simulations is distinct from the requirement that distributions of the statistic from neutral simulations must be generated to reject neutrality at candidate sweep regions. Any sweep statistic, regardless of it being a summary, likelihood, or machine learning approach will require extensive simulations under realistic genetic and evolutionary models to reject the null hypothesis of neutrality.

While optimizing the Λ statistic, we also obtain estimates of the number of presently-sweeping haplotypes *m* and the footprint size *A*. For recent sweeps that are strong enough, estimates of *m* correlate well with the number of initially-selected haplotypes *ν*. For older and less strong sweeps, mutation and recombination events accumulate leading to more distinct haplotypes, thereby inflating *m* estimates. Moreover, estimates of the footprint size *A* correlate with power of Λ, suggesting that the estimated footprint size will be large under scenarios in which sweeps are highly supported. The relationship between *A* and power of Λ is related to prominence of the distortions in the HFS, which also erode due mutation and recombination rates, and this parameter is analogous to the *α* parameter [79] used by other composite likelihood methods to mechanistically model the probability that a lineage escapes a sweep [17, 24]. Therefore, though we found that estimates of *m* were not highly accurate under non-ideal sweep settings and that the precise relationship of *A* to the timing and strength of a sweep is unclear, these quantities may still be useful. Specifically, even if the estimates of *m* are not highly accurate proxies for *ν*, estimates of *m* could still be valuable by casting the problem as binary sweep classification with *m* = 1 for hard and *m* > 1 for soft sweeps, as was also suggested for the *T* statistic by [13]. Table 1 highlights that the *LCT* region is identified as a hard sweep (estimated *m* = 1) in the CEU, with inferred soft sweeps (estimated *m* > 1) in the MHC region, which are consistent with the number of prominent high-frequency haplotypes at these regions (Fig 6). Moreover, though not directly associated with population-genetic parameters such as *ν* or the strength *s* and time *t* of a sweep, estimated Λ, *μ*, and *A* values can be used as input features to machine learning regression algorithms to predict underlying evolutionary model parameters of *ν*, *s*, and *t* [80]. Such strategies are typically computationally expensive, but may be required for accurate characterization of sweep footprints, even though they are unnecessary for detecting sweeps due to the already high power of Λ.

The Λ statistic developed here represents an important step in advancing methodology for sweep detection by interrogating the spatial distribution of distortions in the HFS. Prior studies focused either on spatial distributions of the SFS, which cannot distinguish between hard and soft sweeps, or only local distortions in the HFS. Specifically, methods that explore the skews in the SFS typically do so with an explicit analytical population-genetic model [16, 17, 19–21], which are underpowered if the assumed model is incorrect and are underpowered to detect soft sweeps [78]. In contrast, analytical population-genetic modeling of distortions in the HFS is difficult, and alternative statistical models that capture relevant features of sweeps are often used, focusing either on local distortions in the HFS [13] or haplotype length distributions [5, 7]. Instead, our Λ statistic represents a compromise of these two extremes, permitting simultaneous interrogations of haplotype frequency distributions and correlates of their length distributions in a computationally efficient framework that leads to expected patterns that are informed by theoretical results. Our methodological framework therefore provides a foundation for developing tools that can identify other evolutionary processes that may act locally in the genome, enhancing future investigations of sweeps and other forces across a variety of study systems.

## Methods

In this section we outline the methods used to assess the power of a diversity of sweep statistics using simulations. These simulations examine an array of model parameters, including sweep strength, age, and softness as well as the confounding effects of demographic history, background selection, haplotype phasing, and recombination rate variation. We also describe pre- and post-analysis processing for the application of the Λ statistic to our two real-data examples: CEU and YRI human populations and a rat population from New York City.

### Power analysis

To assess the ability of Λ to detect sweeps, we conducted forward-time simulations using SLiMv3.2 [83] for sweeps of varying strength, age, and softness under a constant-size demographic history as well as under a realistic non-equilibrium demographic history inspired by human studies. Specifically, for each simulation scenario, we generated 1000 independent replicates of length 500 kb, so that Λ was able to interrogate the spatial distribution of variation across a large genomic segment. We employed a mutation rate of *μ* = 1.29 × 10^−8^ per site per generation [84, 85] and a recombination rate of *r* = 10^−8^ per site per generation [86]. For the constant-size demographic history, we considered a population size of *N* = 10^4^ diploid individuals [87], and to investigate complex non-equilibrium demographic histories, we employed the model inferred in [34] of central European humans (CEU), which incorporates a recent bottleneck with a severe population collapse followed by rapid population expansion. In particular, we used this non-equilibrium model as it was inferred by the contemporary method SMC++ [34], which attempts to fit model parameters that can both recapitulate haplotype diversity and allele frequency distributions [88] observed in genomic data from the CEU population of the 1000 Genomes Project dataset [31]. We also considered a setting in which recombination rate was permitted to vary across simulation replicates under the CEU demographic model, with recombination rate for a given simulated replicate drawn from an exponential distribution with mean *r* = 10^−8^ per site per generation (i.e., inspired by [27]).

In addition to these genetic and demographic parameters, for selection simulations, we modeled sweeps on *ν* ∈ {1, 2, 4, 8, 16} initially-selected haplotypes, where each of these haplotypes harbored a beneficial allele in the center of the simulated genomic segment with strength *s* ∈ {0.001, 0.01, 0.1} per generation that immediately appeared and became beneficial at time *t* ∈ {500, 1000, 1500, 2000, 2500, 3000} generations prior to sampling. To ensure that a sweep signature had the potential to be uncovered (especially under settings with *s* = 0.001 and 0.01), we required that the beneficial allele established in the population by reaching a frequency of 0.1 in the population. Simulation replicates for which the beneficial allele did not reach a frequency of 0.1 in the population were repeated until the beneficial allele established in the population. All neutral and selection simulations were run for 11*N* generations, where the first 10*N* generations were used as burn-in and *n* = 50 diploid individuals were sampled from the population after 11*N* generations (*i.e*., the present). Because forward-time simulations are computationally intensive, as is commonly-practiced [89, 90] we scaled all constant-size demographic history simulations by a factor λ = 10 and the European human history by λ = 20, such that the selection coefficient, mutation rate, and recombination rate were multiplied by λ and the population size at each generation and the total number of simulated generations were divided by λ. This scaling leads to a speedup of approximately λ^2^ in computing time, such that the constant-size simulations run roughly 100 times faster than without scaling and the CEU model simulations run approximately 400 times faster, making a large-scale simulation study feasible.

When analyzing each simulated replicate, we examined the performance of Λ with the likelihood *T* statistic [13] that does not account for the spatial distribution of genomic variation, the summary statistic H12 [8] that was developed to detect hard and soft sweeps with similar power, and the standardized iHS [5] and *nS*_*L*_ [7] methods that summarize the lengths of haplotypes centered on core SNPs. When applying one of these sweep detection statistics to a simulated replicate, we scanned the entire simulated region, and the score of the applied statistic for that simulated replicate was chosen as the maximum value of that statistic, computed across all test positions within the simulated region. To investigate the effect of window size on the relative powers of Λ, *T*, and H12, we considered their applications in central windows of 51, 101, and 201 SNPs, and analyzed windows every 25 SNPs across a simulated sequence. We chose SNP-delimited windows rather than windows based on physical length as they should be more robust to variation in recombination and mutation rate across the genome, as well as random missing genomic segments due to poor mappability, alignability, or sequence quality. That is, we expect SNP-delimited to be more conservative than windows based on the physical length of an analyzed genomic segment. We also examined the application of Λ, *T*, and G123 (analogue of H12 [10]) to unphased multilocus genotype input data to evaluate the relative powers of these three approaches when applied on study systems for which obtaining phased haplotypes is difficult, unreliable, or impossible [91]. We applied the lassip software released with this article for application of the saltiLASSI Λ statistic, the LASSI *T* statistic, and H12 (and G123), and the selscan software [90] to compute standardized iHS and *nS*_*L*_.

### Analysis of 1000 Genomes data

We extracted the phased genomes of CEU (99 diploids) and YRI (108 diploids) populations, separately, from the full 1000 Genomes Project Phase 3 dataset (2504 diploids) [31]. For each population, we retained only autosomal biallelic SNPs that were polymorphic in the sample. In order to avoid potentially spurious signals, we also filtered any regions with poor mappability as indicated by mean CRG100 < 0.9 [19, 93]. This left 12,400,078 SNPs in CEU and 20,417,698 SNPs in YRI.

We compute saltiLASSI Λ statistics for both phased (haplotype-based) and unphased (multilocus-genotype-based) analyses with lassip. We use physical distance as the distance measure, and we set --winsize 201, --winstep 100, and --k 20 to use the ranked HFS for the top *K* = 20 most frequent haplotypes. By default lassip assumes phased data and computes haplotype-based statistics, when the --unphased flag is set, all statistics are computed using multilocus genotypes.

To determine significance thresholds, we simulated neutral whole genomes with a realistic recombination map and demographic history using stdpopsim [85] and msprime [94]. Using the OutOfAfrica_2T12 demographic history [95] and the HapMapII_GRCh37 genetic map [96] in stdpopsim, we simulate 100 replicates of all 22 autosomes for each population separately, sampling 99 diploid individuals for CEU simulations and 108 diploid individuals for YRI simulations. For each replicate, we then compute saltiLASSI Λ statistics for both phased and unphased analyses with lassip, setting --winsize 201, --winstep 100, and --k 20. As simulated genomes do not simulate variants at the same sites, the windows within which Λ is calculated will not perfectly align with each other or our real-data analysis. In order to compare neutral and real Λ values at local regions of the genome, for each neutral replicate, separately, we align the simulated windows to the windows of our real-data analysis, and then for each real-data window we calculate a weighted mean of all overlapping windows to get a neutral-simulation Λ for that window associated with our real-data. In this way we are able to compute 100 neutral-simulated Λ values for each window in our real-data analyses. We then compute the max Λ, the top-0.1% Λ, and the top-1% Λ across all windows in all replicates for each population and each analysis (phased/unphased), which are given in S1 Table. We consider any window with a Λ greater than the max observed across all genome analysis windows from all neutral simulations as a putatively selected region, and we concatenate consecutive windows satisfying this condition into larger regions implicated as being under selection (phased in Tables 1 and 2 and unphased in S5 and S6 Tables).

Finally, we also compute Λ for all simulated and empirical data using two other distance measures: number of windows and centiMorgans. For the latter measure we use the HapMapII_GRCh37 genetic map [96] and use the genetic distance between window midpoints. Midpoints for which a genetic position does not exist in the HapMapII_GRCh37 genetic map are linearly interpolated based on the nearest surrounding sites. We compare these results to the results calculated using physical distance using Spearman’s rank correlation (S4 Table). For simulated data, we compute the mean correlation coefficient across all 100 replicates.

### Analysis of New York City rats

We extracted the genetic data of 29 rats sampled in New York City [32], retaining only autosomal biallelic SNPs that were polymorphic in the sample. This left 13,532,711 SNPs. As these data are unphased, we use lassip to compute saltiLASSI Λ statistic using multilocus-genotypes (--unphased flag). We set --winsize 201 and --winstep 100, and we choose --k 20 to use the ranked HFS for the top *K* = 20 most frequent haplotypes.

[32] noted that the demographic history for brown rats was likely poorly calibrated for these New York City samples. We therefore take an outlier approach for analyzing the results of the saltiLASSI method on these data. We compute the top-0.1% Λ, the top-1% Λ, and the top-5% Λ across all windows genome-wide, getting 389.839, 88.080, and 22.724, respectively. Putatively selected regions were identified by concatenating consecutive windows with Λ greater than the top-1% Λ observed (S7 and S8 Tables). The 1000 Genomes Project data is available at http://ftp.1000genomes.ebi.ac.uk/vol1/ftp/release/20130502/, and the New York City rat data is available at https://doi.org/10.5061/dryad.08kprr4zn. Analysis scripts and intermediate data files used in this study are available from Data Dryad at doi:10.5061/dryad.4qrfj6qbm [81, 82].

## Dryad DOI

https://doi.org/10.5061/dryad.08kprr4zn.

doi:10.5061/dryad.4qrfj6qbm.

## Supporting information

S1 Fig Power at a 1% false positive rate (FPR). As a function of selection start time for applications of Λ, *T*, and H12 with windows of size 51, 101, and 201 SNPs, as well *nS*_*L*_ and iHS under simulations of a constant-size demographic history for per-generation selection coefficients of *s* ∈ {0.001, 0.01, 0.1} on the rows. Classification ability demonstrated for selection start times of *t* ∈ {500, 1000, 1500, 2000, 2500, 3000} generations prior to sampling for *ν* ∈ {1, 2, 4, 8, 16} initially-selected haplotypes (columns). Results are based on a sample of *n* = 50 diploid individuals and the haplotype frequency spectra for the Λ and *T* statistics truncated at *K* = 10 haplotypes.
(EPS)

S2 FigEstimated sweep softness.Illustrated by mean estimated number of sweeping haplotypes ($\hat{m}$) in Λ with windows of size 51, 101, and 201 SNPs under simulations of a constant-size demographic history for per-generation selection coefficients of *s* ∈ {0.001, 0.01, 0.1} on the rows. Mean estimated softness demonstrated for selection start times of *t* ∈ {500, 1000, 1500, 2000, 2500, 3000} generations prior to sampling for *ν* ∈ {1, 2, 4, 8, 16} initially-selected haplotypes (columns). Gray solid, dashed, and dotted horizontal lines are the corresponding mean $\hat{m}$ values for Λ applied to neutral simulations. Results are based on a sample of *n* = 50 diploid individuals and the haplotype frequency spectrum for the Λ statistic truncated at *K* = 10 haplotypes.(EPS)

S3 FigEstimated sweep width.Illustrated by mean estimated genomic size influenced by the sweep (${\text{log}}_{10}\;\hat{A}$) in Λ with windows of size 51, 101, and 201 SNPs under simulations of a constant-size demographic history for per-generation selection coefficients of *s* ∈ {0.001, 0.01, 0.1} on the rows. Mean estimated genomic size influenced by sweeps demonstrated for selection start times of *t* ∈ {500, 1000, 1500, 2000, 2500, 3000} generations prior to sampling for *ν* ∈ {1, 2, 4, 8, 16} initially-selected haplotypes (columns). Gray solid, dashed, and dotted horizontal lines are the corresponding mean ${\text{log}}_{10}\;\hat{A}$ values for Λ applied to neutral simulations. Results are based on a sample of *n* = 50 diploid individuals and the haplotype frequency spectrum for the Λ statistic truncated at *K* = 10 haplotypes.(EPS)

S4 Fig Power at a 1% false positive rate (FPR). As a function of selection start time for applications of Λ, *T*, and G123 with windows of size 51, 101, and 201 SNPs to unphased multilocus genotype input data under simulations of a constant-size demographic history for per-generation selection coefficients of *s* ∈ {0.001, 0.01, 0.1} on the rows. Classification ability demonstrated for selection start times of *t* ∈ {500, 1000, 1500, 2000, 2500, 3000} generations prior to sampling for *ν* ∈ {1, 2, 4, 8, 16} initially-selected haplotypes (columns). Results are based on a sample of *n* = 50 diploid individuals and the multilocus genotype frequency spectra for the Λ and *T* statistics truncated at *K* = 10 multilocus genotypes.
(EPS)

S5 FigEstimated sweep softness.Illustrated by mean estimated number of sweeping haplotypes ($\hat{m}$) in Λ with windows of size 51, 101, and 201 SNPs applied to unphased multilocus input data under simulations of a constant-size demographic history for per-generation selection coefficients of *s* ∈ {0.001, 0.01, 0.1} on the rows. Mean estimated softness demonstrated for selection start times of *t* ∈ {500, 1000, 1500, 2000, 2500, 3000} generations prior to sampling for *ν* ∈ {1, 2, 4, 8, 16} initially-selected haplotypes (columns). Gray solid, dashed, and dotted horizontal lines are the corresponding mean $\hat{m}$ values for Λ applied to neutral simulations. Results are based on a sample of *n* = 50 diploid individuals and the multilocus genotype frequency spectrum for the Λ statistic truncated at *K* = 10 multilocus genotypes.(EPS)

S6 FigEstimated sweep width.Illustrated by mean estimated genomic size influenced by the sweep (${\text{log}}_{10}\;\hat{A}$) in Λ with windows of size 51, 101, and 201 SNPs applied to unphased multilocus input data under simulations of a constant-size demographic history for per-generation selection coefficients of *s* ∈ {0.001, 0.01, 0.1} on the rows. Mean estimated genomic size influenced by sweeps demonstrated for selection start times of *t* ∈ {500, 1000, 1500, 2000, 2500, 3000} generations prior to sampling for *ν* ∈ {1, 2, 4, 8, 16} initially-selected haplotypes (columns). Gray solid, dashed, and dotted horizontal lines are the corresponding mean ${\text{log}}_{10}\;\hat{A}$ values for Λ applied to neutral simulations. Results are based on a sample of *n* = 50 diploid individuals and the multilocus genotype frequency spectrum for the Λ statistic truncated at *K* = 10 multilocus genotypes.(EPS)

S7 Fig Power at a 1% false positive rate (FPR). As a function of selection start time for applications of Λ, *T*, and H12 with windows of size 51, 101, and 201 SNPs, as well *nS*_*L*_ and iHS under simulations of the human central European (CEU) demographic history of [34] for per-generation selection coefficients of *s* ∈ {0.001, 0.01, 0.1} on the rows. Classification ability demonstrated for selection start times of *t* ∈ {500, 1000, 1500, 2000, 2500, 3000} generations prior to sampling for *ν* ∈ {1, 2, 4, 8, 16} initially-selected haplotypes (columns). Results are based on a sample of *n* = 50 diploid individuals and the haplotype frequency spectra for the Λ and *T* statistics truncated at *K* = 10 haplotypes.
(EPS)

S8 FigEstimated sweep softness.Illustrated by mean estimated number of sweeping haplotypes ($\hat{m}$) in Λ with windows of size 51, 101, and 201 SNPs under simulations of the human central European (CEU) demographic history of [34] for per-generation selection coefficients of *s* ∈ {0.001, 0.01, 0.1} on the rows. Mean estimated softness demonstrated for selection start times of *t* ∈ {500, 1000, 1500, 2000, 2500, 3000} generations prior to sampling for *ν* ∈ {1, 2, 4, 8, 16} initially-selected haplotypes (columns). Gray solid, dashed, and dotted horizontal lines are the corresponding mean $\hat{m}$ values for Λ applied to neutral simulations. Results are based on a sample of *n* = 50 diploid individuals and the haplotype frequency spectrum for the Λ statistic truncated at *K* = 10 haplotypes.(EPS)

S9 FigEstimated sweep width.Illustrated by mean estimated genomic size influenced by the sweep (${\text{log}}_{10}\;\hat{A}$) in Λ with windows of size 51, 101, and 201 SNPs under simulations of the human central European (CEU) demographic history of [34] for per-generation selection coefficients of *s* ∈ {0.001, 0.01, 0.1} on the rows. Mean estimated genomic size influenced by sweeps demonstrated for selection start times of *t* ∈ {500, 1000, 1500, 2000, 2500, 3000} generations prior to sampling for *ν* ∈ {1, 2, 4, 8, 16} initially-selected haplotypes (columns). Gray solid, dashed, and dotted horizontal lines are the corresponding mean ${\text{log}}_{10}\;\hat{A}$ values for Λ applied to neutral simulations. Results are based on a sample of *n* = 50 diploid individuals and the haplotype frequency spectrum for the Λ statistic truncated at *K* = 10 haplotypes.(EPS)

S10 Fig Power at a 1% false positive rate (FPR). As a function of selection start time for applications of Λ, *T*, and G123 with windows of size 51, 101, and 201 SNPs to unphased multilocus genotype input data under simulations of the human central European (CEU) demographic history of [34] for per-generation selection coefficients of *s* ∈ {0.001, 0.01, 0.1} on the rows. Classification ability demonstrated for selection start times of *t* ∈ {500, 1000, 1500, 2000, 2500, 3000} generations prior to sampling for *ν* ∈ {1, 2, 4, 8, 16} initially-selected haplotypes (columns). Results are based on a sample of *n* = 50 diploid individuals and the multilocus genotype frequency spectra for the Λ and *T* statistics truncated at *K* = 10 multilocus genotypes.
(EPS)

S11 FigEstimated sweep softness.Illustrated by mean estimated number of sweeping haplotypes ($\hat{m}$) in Λ with windows of size 51, 101, and 201 SNPs applied to unphased multilocus input data under simulations of the human central European (CEU) demographic history of [34] for per-generation selection coefficients of *s* ∈ {0.001, 0.01, 0.1} on the rows. Mean estimated softness demonstrated for selection start times of *t* ∈ {500, 1000, 1500, 2000, 2500, 3000} generations prior to sampling for *ν* ∈ {1, 2, 4, 8, 16} initially-selected haplotypes (columns). Gray solid, dashed, and dotted horizontal lines are the corresponding mean $\hat{m}$ values for Λ applied to neutral simulations. Results are based on a sample of *n* = 50 diploid individuals and the multilocus genotype frequency spectrum for the Λ statistic truncated at *K* = 10 multilocus genotypes.(EPS)

S12 FigEstimated sweep width.Illustrated by mean estimated genomic size influenced by the sweep (${\text{log}}_{10}\;\hat{A}$) in Λ with windows of size 51, 101, and 201 SNPs applied to unphased multilocus input data under simulations of the human central European (CEU) demographic history of [34] for per-generation selection coefficients of *s* ∈ {0.001, 0.01, 0.1} on the rows. Mean estimated genomic size influenced by sweeps demonstrated for selection start times of *t* ∈ {500, 1000, 1500, 2000, 2500, 3000} generations prior to sampling for *ν* ∈ {1, 2, 4, 8, 16} initially-selected haplotypes (columns). Gray solid, dashed, and dotted horizontal lines are the corresponding mean ${\text{log}}_{10}\;\hat{A}$ values for Λ applied to neutral simulations. Results are based on a sample of *n* = 50 diploid individuals and the multilocus genotype frequency spectrum for the Λ statistic truncated at *K* = 10 multilocus genotypes.(EPS)

S13 Fig Power at a 1% false positive rate (FPR). As a function of selection start time for applications of Λ, *T*, and H12 with windows of size 51, 101, and 201 SNPs, as well *nS*_*L*_ and iHS under simulations of the human central European (CEU) demographic history of [34] with per-site per-generation recombination rate drawn from an exponential distribution with mean of 10^−8^ for per-generation selection coefficients of *s* ∈ {0.001, 0.01, 0.1} on the rows. Classification ability demonstrated for selection start times of *t* ∈ {500, 1000, 1500, 2000, 2500, 3000} generations prior to sampling for *ν* ∈ {1, 2, 4, 8, 16} initially-selected haplotypes (columns). Results are based on a sample of *n* = 50 diploid individuals and the haplotype frequency spectra for the Λ and *T* statistics truncated at *K* = 10 haplotypes. Plots displaying patterns in estimated sweep softness and footprint size can be found in S14 and S15 Figs, respectively.
(EPS)

S14 FigEstimated sweep softness.Illustrated by mean estimated number of sweeping haplotypes ($\hat{m}$) in Λ with windows of size 51, 101, and 201 SNPs under simulations of the human central European (CEU) demographic history of [34] with per-site per-generation recombination rate drawn from an exponential distribution with mean of 10^−8^ for per-generation selection coefficients of *s* ∈ {0.001, 0.01, 0.1} on the rows. Mean estimated softness demonstrated for selection start times of *t* ∈ {500, 1000, 1500, 2000, 2500, 3000} generations prior to sampling for *ν* ∈ {1, 2, 4, 8, 16} initially-selected haplotypes (columns). Gray solid, dashed, and dotted horizontal lines are the corresponding mean $\hat{m}$ values for Λ applied to neutral simulations. Results are based on a sample of *n* = 50 diploid individuals and the haplotype frequency spectrum for the Λ statistic truncated at *K* = 10 haplotypes.(EPS)

S15 FigEstimated sweep width.Illustrated by mean estimated genomic size influenced by the sweep (${\text{log}}_{10}\;\hat{A}$) in Λ with windows of size 51, 101, and 201 SNPs under simulations of the human central European (CEU) demographic history of [34] with per-site per-generation recombination rate drawn from an exponential distribution with mean of 10^−8^ for per-generation selection coefficients of *s* ∈ {0.001, 0.01, 0.1} on the rows. Mean estimated genomic size influenced by sweeps demonstrated for selection start times of *t* ∈ {500, 1000, 1500, 2000, 2500, 3000} generations prior to sampling for *ν* ∈ {1, 2, 4, 8, 16} initially-selected haplotypes (columns). Gray solid, dashed, and dotted horizontal lines are the corresponding mean ${\text{log}}_{10}\;\hat{A}$ values for Λ applied to neutral simulations. Results are based on a sample of *n* = 50 diploid individuals and the haplotype frequency spectrum for the Λ statistic truncated at *K* = 10 haplotypes.(EPS)

S16 Fig Power at a 1% false positive rate (FPR). As a function of selection start time for applications of Λ, *T*, and G123 with windows of size 51, 101, and 201 SNPs to unphased multilocus genotype input data under simulations of the human central European (CEU) demographic history of [34] with per-site per-generation recombination rate drawn from an exponential distribution with mean of 10^−8^ for per-generation selection coefficients of *s* ∈ {0.001, 0.01, 0.1} on the rows. Classification ability demonstrated for selection start times of *t* ∈ {500, 1000, 1500, 2000, 2500, 3000} generations prior to sampling for *ν* ∈ {1, 2, 4, 8, 16} initially-selected haplotypes (columns). Results are based on a sample of *n* = 50 diploid individuals and the multilocus genotype frequency spectra for the Λ and *T* statistics truncated at *K* = 10 multilocus genotypes. Plots displaying patterns in estimated sweep softness and footprint size can be found in S17 and S18 Figs, respectively.
(EPS)

S17 FigEstimated sweep softness.Illustrated by mean estimated number of sweeping haplotypes ($\hat{m}$) in Λ with windows of size 51, 101, and 201 SNPs applied to unphased multilocus input data under simulations of the human central European (CEU) demographic history of [34] with per-site per-generation recombination rate drawn from an exponential distribution with mean of 10^−8^ for per-generation selection coefficients of *s* ∈ {0.001, 0.01, 0.1} on the rows. Mean estimated softness demonstrated for selection start times of *t* ∈ {500, 1000, 1500, 2000, 2500, 3000} generations prior to sampling for *ν* ∈ {1, 2, 4, 8, 16} initially-selected haplotypes (columns). Gray solid, dashed, and dotted horizontal lines are the corresponding mean $\hat{m}$ values for Λ applied to neutral simulations. Results are based on a sample of *n* = 50 diploid individuals and the multilocus genotype frequency spectrum for the Λ statistic truncated at *K* = 10 multilocus genotypes.(EPS)

S18 FigEstimated sweep width.Illustrated by mean estimated genomic size influenced by the sweep (${\text{log}}_{10}\;\hat{A}$) in Λ with windows of size 51, 101, and 201 SNPs applied to unphased multilocus input data under simulations of the human central European (CEU) demographic history of [34] with per-site per-generation recombination rate drawn from an exponential distribution with mean of 10^−8^ for per-generation selection coefficients of *s* ∈ {0.001, 0.01, 0.1} on the rows. Mean estimated genomic size influenced by sweeps demonstrated for selection start times of *t* ∈ {500, 1000, 1500, 2000, 2500, 3000} generations prior to sampling for *ν* ∈ {1, 2, 4, 8, 16} initially-selected haplotypes (columns). Gray solid, dashed, and dotted horizontal lines are the corresponding mean ${\text{log}}_{10}\;\hat{A}$ values for Λ applied to neutral simulations. Results are based on a sample of *n* = 50 diploid individuals and the multilocus genotype frequency spectrum for the Λ statistic truncated at *K* = 10 multilocus genotypes.(EPS)

S19 Fig Proportion of false signals. As a function of false positive rate for applications of Λ, *T*, H12, and G123 with windows of size 51, 101, and 201 SNPs, as well *nS*_*L*_ and iHS under simulations of a constant-size demographic history and the human central European (CEU) demographic history of [34] (bottleneck scenario) under background selection using either phased haplotype input data (Λ, *T*, H12, *nS*_*L*_, and iHS) or unphased multilocus genotype input data (Λ, *T*, and G123). Proportion of false signals is computed as the fraction of background selection simulations in which the score computed for Λ, *T*, H12, G123, *nS*_*L*_, or iHS exceeded the corresponding score threshold defined by a particular false positive rate. Results are based on a sample of *n* = 50 diploid individuals and haplotype and multilocus genotype frequency spectra for the Λ and *T* statistics truncated at *K* = 10 haplotypes or multilocus genotypes.
(EPS)

S20 Fig Power at a 1% false positive rate (FPR). as a function of selection start time for applications of Λ with windows of size 51, 101, and 201 SNPs under simulations of a constant-size demographic history and sample size of *n* ∈ {10, 25, 50} diploid individuals for per-generation selection coefficients of *s* ∈ {0.001, 0.01, 0.1} on the rows. Classification ability demonstrated for selection start times of *t* ∈ {500, 1000, 1500, 2000, 2500, 3000} generations prior to sampling for *ν* ∈ {1, 2, 4, 8, 16} initially-selected haplotypes (columns). Results are based on the haplotype frequency spectra for the Λ statistics truncated at *K* = 10 haplotypes.
(EPS)

S21 FigEstimated sweep softness.Illustrated by mean estimated number of sweeping haplotypes ($\hat{m}$) in Λ with windows of size 51, 101, and 201 SNPs under simulations of a constant-size demographic history and sample size of *n* ∈ {10, 25, 50} diploid individuals for per-generation selection coefficients of *s* ∈ {0.001, 0.01, 0.1} on the rows. Mean estimated softness demonstrated for selection start times of *t* ∈ {500, 1000, 1500, 2000, 2500, 3000} generations prior to sampling for *ν* ∈ {1, 2, 4, 8, 16} initially-selected haplotypes (columns). Results are based on the haplotype frequency spectrum for the Λ statistic truncated at *K* = 10 haplotypes.(EPS)

S22 FigEstimated sweep width.Illustrated by mean estimated genomic size influenced by the sweep (${\text{log}}_{10}\;\hat{A}$) in Λ with windows of size 51, 101, and 201 SNPs under simulations of a constant-size demographic history and sample size of *n* ∈ {10, 25, 50} diploid individuals for per-generation selection coefficients of *s* ∈ {0.001, 0.01, 0.1} on the rows. Mean estimated genomic size influenced by sweeps demonstrated for selection start times of *t* ∈ {500, 1000, 1500, 2000, 2500, 3000} generations prior to sampling for *ν* ∈ {1, 2, 4, 8, 16} initially-selected haplotypes (columns). Results are based on the haplotype frequency spectrum for the Λ statistic truncated at *K* = 10 haplotypes.(EPS)

S23 Fig Power at a 1% false positive rate (FPR). As a function of selection start time for applications of Λ with windows of size 51, 101, and 201 SNPs to unphased multilocus genotype input data under simulations of a constant-size demographic history and sample size of *n* ∈ {10, 25, 50} diploid individuals for per-generation selection coefficients of *s* ∈ {0.001, 0.01, 0.1} on the rows. Classification ability demonstrated for selection start times of *t* ∈ {500, 1000, 1500, 2000, 2500, 3000} generations prior to sampling for *ν* ∈ {1, 2, 4, 8, 16} initially-selected haplotypes (columns). Results are based on the multilocus genotype frequency spectrum for the Λ statistic truncated at *K* = 10 multilocus genotypes.
(EPS)

S24 FigEstimated sweep softness.Illustrated by mean estimated number of sweeping haplotypes ($\hat{m}$) in Λ with windows of size 51, 101, and 201 SNPs applied to unphased multilocus input data under simulations of a constant-size demographic history and sample size of *n* ∈ {10, 25, 50} diploid individuals for per-generation selection coefficients of *s* ∈ {0.001, 0.01, 0.1} on the rows. Mean estimated softness demonstrated for selection start times of *t* ∈ {500, 1000, 1500, 2000, 2500, 3000} generations prior to sampling for *ν* ∈ {1, 2, 4, 8, 16} initially-selected haplotypes (columns). Results are based on the multilocus genotype frequency spectrum for the Λ statistic truncated at *K* = 10 multilocus genotypes.(EPS)

S25 FigEstimated sweep width.Illustrated by mean estimated genomic size influenced by the sweep (${\text{log}}_{10}\;\hat{A}$) in Λ with windows of size 51, 101, and 201 SNPs applied to unphased multilocus input data under simulations of a constant-size demographic history and sample size of *n* ∈ {10, 25, 50} diploid individuals for per-generation selection coefficients of *s* ∈ {0.001, 0.01, 0.1} on the rows. Mean estimated genomic size influenced by sweeps demonstrated for selection start times of *t* ∈ {500, 1000, 1500, 2000, 2500, 3000} generations prior to sampling for *ν* ∈ {1, 2, 4, 8, 16} initially-selected haplotypes (columns). Results are based on the multilocus genotype frequency spectrum for the Λ statistic truncated at *K* = 10 multilocus genotypes.(EPS)

S26 Fig Power at a 1% false positive rate (FPR). As a function of selection start time for applications of Λ with windows of size 51, 101, and 201 SNPs under simulations of the human central European (CEU) demographic history of [34] and sample size of *n* ∈ {10, 25, 50} diploid individuals for per-generation selection coefficients of *s* ∈ {0.001, 0.01, 0.1} on the rows. Classification ability demonstrated for selection start times of *t* ∈ {500, 1000, 1500, 2000, 2500, 3000} generations prior to sampling for *ν* ∈ {1, 2, 4, 8, 16} initially-selected haplotypes (columns). Results are based on the haplotype frequency spectra for the Λ statistics truncated at *K* = 10 haplotypes.
(EPS)

S27 FigEstimated sweep softness.Illustrated by mean estimated number of sweeping haplotypes ($\hat{m}$) in Λ with windows of size 51, 101, and 201 SNPs under simulations of the human central European (CEU) demographic history of [34] and sample size of *n* ∈ {10, 25, 50} diploid individuals for per-generation selection coefficients of *s* ∈ {0.001, 0.01, 0.1} on the rows. Mean estimated softness demonstrated for selection start times of *t* ∈ {500, 1000, 1500, 2000, 2500, 3000} generations prior to sampling for *ν* ∈ {1, 2, 4, 8, 16} initially-selected haplotypes (columns). Results are based on the haplotype frequency spectrum for the Λ statistic truncated at *K* = 10 haplotypes.(EPS)

S28 FigEstimated sweep width.Illustrated by mean estimated genomic size influenced by the sweep (${\text{log}}_{10}\;\hat{A}$) in Λ with windows of size 51, 101, and 201 SNPs under simulations of the human central European (CEU) demographic history of [34] and sample size of *n* ∈ {10, 25, 50} diploid individuals for per-generation selection coefficients of *s* ∈ {0.001, 0.01, 0.1} on the rows. Mean estimated genomic size influenced by sweeps demonstrated for selection start times of *t* ∈ {500, 1000, 1500, 2000, 2500, 3000} generations prior to sampling for *ν* ∈ {1, 2, 4, 8, 16} initially-selected haplotypes (columns). Results are based on the haplotype frequency spectrum for the Λ statistic truncated at *K* = 10 haplotypes.(EPS)

S29 Fig Power at a 1% false positive rate (FPR). As a function of selection start time for applications of Λ with windows of size 51, 101, and 201 SNPs to unphased multilocus genotype input data under simulations of the human central European (CEU) demographic history of [34] and sample size of *n* ∈ {10, 25, 50} diploid individuals for per-generation selection coefficients of *s* ∈ {0.001, 0.01, 0.1} on the rows. Classification ability demonstrated for selection start times of *t* ∈ {500, 1000, 1500, 2000, 2500, 3000} generations prior to sampling for *ν* ∈ {1, 2, 4, 8, 16} initially-selected haplotypes (columns). Results are based on the multilocus genotype frequency spectrum for the Λ statistic truncated at *K* = 10 multilocus genotypes.
(EPS)

S30 FigEstimated sweep softness.Illustrated by mean estimated number of sweeping haplotypes ($\hat{m}$) in Λ with windows of size 51, 101, and 201 SNPs applied to unphased multilocus input data under simulations of the human central European (CEU) demographic history of [34] and sample size of *n* ∈ {10, 25, 50} diploid individuals for per-generation selection coefficients of *s* ∈ {0.001, 0.01, 0.1} on the rows. Mean estimated softness demonstrated for selection start times of *t* ∈ {500, 1000, 1500, 2000, 2500, 3000} generations prior to sampling for *ν* ∈ {1, 2, 4, 8, 16} initially-selected haplotypes (columns). Results are based on the multilocus genotype frequency spectrum for the Λ statistic truncated at *K* = 10 multilocus genotypes.(EPS)

S31 FigEstimated sweep width.Illustrated by mean estimated genomic size influenced by the sweep (${\text{log}}_{10}\;\hat{A}$) in Λ with windows of size 51, 101, and 201 SNPs applied to unphased multilocus input data under simulations of the human central European (CEU) demographic history of [34] and sample size of *n* ∈ {10, 25, 50} diploid individuals for per-generation selection coefficients of *s* ∈ {0.001, 0.01, 0.1} on the rows. Mean estimated genomic size influenced by sweeps demonstrated for selection start times of *t* ∈ {500, 1000, 1500, 2000, 2500, 3000} generations prior to sampling for *ν* ∈ {1, 2, 4, 8, 16} initially-selected haplotypes (columns). Results are based on the multilocus genotype frequency spectrum for the Λ statistic truncated at *K* = 10 multilocus genotypes.(EPS)

S32 Fig Power at a 1% false positive rate (FPR). As a function of selection start time for applications of Λ with windows of size 51, 101, and 201 SNPs under simulations of a constant-size demographic history and the haplotype frequency spectra for the Λ statistic truncated at *K* ∈ {5, 10, 20} haplotypes for per-generation selection coefficients of *s* ∈ {0.001, 0.01, 0.1} on the rows. Classification ability demonstrated for selection start times of *t* ∈ {500, 1000, 1500, 2000, 2500, 3000} generations prior to sampling for *ν* ∈ {1, 2, 4, 8, 16} initially-selected haplotypes (columns). Results are based on a sample of *n* = 50 diploid individuals.
(EPS)

S33 FigEstimated sweep softness.Illustrated by mean estimated number of sweeping haplotypes ($\hat{m}$) in Λ with windows of size 51, 101, and 201 SNPs under simulations of a constant-size demographic history and the haplotype frequency spectra for the Λ statistic truncated at *K* ∈ {5, 10, 20} haplotypes for per-generation selection coefficients of *s* ∈ {0.001, 0.01, 0.1} on the rows. Mean estimated softness demonstrated for selection start times of *t* ∈ {500, 1000, 1500, 2000, 2500, 3000} generations prior to sampling for *ν* ∈ {1, 2, 4, 8, 16} initially-selected haplotypes (columns). Results are based on a sample of *n* = 50 diploid individuals.(EPS)

S34 FigEstimated sweep width.Illustrated by mean estimated genomic size influenced by the sweep (${\text{log}}_{10}\;\hat{A}$) in Λ with windows of size 51, 101, and 201 SNPs under simulations of a constant-size demographic history and the haplotype frequency spectra for the Λ statistic truncated at *K* ∈ {5, 10, 20} haplotypes for per-generation selection coefficients of *s* ∈ {0.001, 0.01, 0.1} on the rows. Mean estimated genomic size influenced by sweeps demonstrated for selection start times of *t* ∈ {500, 1000, 1500, 2000, 2500, 3000} generations prior to sampling for *ν* ∈ {1, 2, 4, 8, 16} initially-selected haplotypes (columns). Results are based on a sample of *n* = 50 diploid individuals.(EPS)

S35 Fig Power at a 1% false positive rate (FPR). As a function of selection start time for applications of Λ with windows of size 51, 101, and 201 SNPs to unphased multilocus genotype input data under simulations of a constant-size demographic history and the multilocus genotype frequency spectra for the Λ statistic truncated at *K* ∈ {5, 10, 20} multilocus genotypes for per-generation selection coefficients of *s* ∈ {0.001, 0.01, 0.1} on the rows. Classification ability demonstrated for selection start times of *t* ∈ {500, 1000, 1500, 2000, 2500, 3000} generations prior to sampling for *ν* ∈ {1, 2, 4, 8, 16} initially-selected haplotypes (columns). Results are based on a sample of *n* = 50 diploid individuals.
(EPS)

S36 FigEstimated sweep softness.Illustrated by mean estimated number of sweeping haplotypes ($\hat{m}$) in Λ with windows of size 51, 101, and 201 SNPs applied to unphased multilocus input data under simulations of a constant-size demographic history and the multilocus genotype frequency spectra for the Λ statistic truncated at *K* ∈ {5, 10, 20} multilocus genotypes for per-generation selection coefficients of *s* ∈ {0.001, 0.01, 0.1} on the rows. Mean estimated softness demonstrated for selection start times of *t* ∈ {500, 1000, 1500, 2000, 2500, 3000} generations prior to sampling for *ν* ∈ {1, 2, 4, 8, 16} initially-selected haplotypes (columns). Results are based on a sample of *n* = 50 diploid individuals.(EPS)

S37 FigEstimated sweep width.Illustrated by mean estimated genomic size influenced by the sweep (${\text{log}}_{10}\;\hat{A}$) in Λ with windows of size 51, 101, and 201 SNPs applied to unphased multilocus input data under simulations of a constant-size demographic history and the multilocus genotype frequency spectra for the Λ statistic truncated at *K* ∈ {5, 10, 20} multilocus genotypes for per-generation selection coefficients of *s* ∈ {0.001, 0.01, 0.1} on the rows. Mean estimated genomic size influenced by sweeps demonstrated for selection start times of *t* ∈ {500, 1000, 1500, 2000, 2500, 3000} generations prior to sampling for *ν* ∈ {1, 2, 4, 8, 16} initially-selected haplotypes (columns). Results are based on a sample of *n* = 50 diploid individuals.(EPS)

S38 Fig Power at a 1% false positive rate (FPR). As a function of selection start time for applications of Λ with windows of size 51, 101, and 201 SNPs under simulations of the human central European (CEU) demographic history of [34] and the haplotype frequency spectra for the Λ statistic truncated at *K* ∈ {5, 10, 20} haplotypes for per-generation selection coefficients of *s* ∈ {0.001, 0.01, 0.1} on the rows. Classification ability demonstrated for selection start times of *t* ∈ {500, 1000, 1500, 2000, 2500, 3000} generations prior to sampling for *ν* ∈ {1, 2, 4, 8, 16} initially-selected haplotypes (columns). Results are based on a sample of *n* = 50 diploid individuals.
(EPS)

S39 FigEstimated sweep softness.Illustrated by mean estimated number of sweeping haplotypes ($\hat{m}$) in Λ with windows of size 51, 101, and 201 SNPs under simulations of the human central European (CEU) demographic history of [34] and the haplotype frequency spectra for the Λ statistic truncated at *K* ∈ {5, 10, 20} haplotypes for per-generation selection coefficients of *s* ∈ {0.001, 0.01, 0.1} on the rows. Mean estimated softness demonstrated for selection start times of *t* ∈ {500, 1000, 1500, 2000, 2500, 3000} generations prior to sampling for *ν* ∈ {1, 2, 4, 8, 16} initially-selected haplotypes (columns). Results are based on a sample of *n* = 50 diploid individuals.(EPS)

S40 FigEstimated sweep width.Illustrated by mean estimated genomic size influenced by the sweep (${\text{log}}_{10}\;\hat{A}$) in Λ with windows of size 51, 101, and 201 SNPs under simulations of the human central European (CEU) demographic history of [34] and the haplotype frequency spectra for the Λ statistic truncated at *K* ∈ {5, 10, 20} haplotypes for per-generation selection coefficients of *s* ∈ {0.001, 0.01, 0.1} on the rows. Mean estimated genomic size influenced by sweeps demonstrated for selection start times of *t* ∈ {500, 1000, 1500, 2000, 2500, 3000} generations prior to sampling for *ν* ∈ {1, 2, 4, 8, 16} initially-selected haplotypes (columns). Results are based on a sample of *n* = 50 diploid individuals.(EPS)

S41 Fig Power at a 1% false positive rate (FPR). As a function of selection start time for applications of Λ with windows of size 51, 101, and 201 SNPs to unphased multilocus genotype input data under simulations of the human central European (CEU) demographic history of [34] and the multilocus genotype frequency spectra for the Λ statistic truncated at *K* ∈ {5, 10, 20} multilocus genotypes for per-generation selection coefficients of *s* ∈ {0.001, 0.01, 0.1} on the rows. Classification ability demonstrated for selection start times of *t* ∈ {500, 1000, 1500, 2000, 2500, 3000} generations prior to sampling for *ν* ∈ {1, 2, 4, 8, 16} initially-selected haplotypes (columns). Results are based on a sample of *n* = 50 diploid individuals.
(EPS)

S42 FigEstimated sweep softness.Illustrated by mean estimated number of sweeping haplotypes ($\hat{m}$) in Λ with windows of size 51, 101, and 201 SNPs applied to unphased multilocus input data under simulations of the human central European (CEU) demographic history of [34] and the multilocus genotype frequency spectra for the Λ statistic truncated at *K* ∈ {5, 10, 20} multilocus genotypes for per-generation selection coefficients of *s* ∈ {0.001, 0.01, 0.1} on the rows. Mean estimated softness demonstrated for selection start times of *t* ∈ {500, 1000, 1500, 2000, 2500, 3000} generations prior to sampling for *ν* ∈ {1, 2, 4, 8, 16} initially-selected haplotypes (columns). Results are based on a sample of *n* = 50 diploid individuals.(EPS)

S43 FigEstimated sweep width.Illustrated by mean estimated genomic size influenced by the sweep (${\text{log}}_{10}\;\hat{A}$) in Λ with windows of size 51, 101, and 201 SNPs applied to unphased multilocus input data under simulations of the human central European (CEU) demographic history of [34] and the multilocus genotype frequency spectra for the Λ statistic truncated at *K* ∈ {5, 10, 20} multilocus genotypes for per-generation selection coefficients of *s* ∈ {0.001, 0.01, 0.1} on the rows. Mean estimated genomic size influenced by sweeps demonstrated for selection start times of *t* ∈ {500, 1000, 1500, 2000, 2500, 3000} generations prior to sampling for *ν* ∈ {1, 2, 4, 8, 16} initially-selected haplotypes (columns). Results are based on a sample of *n* = 50 diploid individuals.(EPS)

S44 Fig Manhattan plot of unphased multi-locus genotype Λ-statistics. For the (A) CEU and (B) YRI populations from the 1000 Genomes Project. Each point represents a single 201-SNP window along the genome. Horizontal lines represent the top 1%, top 0.1%, and maximum observed Λ statistic across all windows in demography-matched neutral simulations. Red line indicates the maximum observed Λ among 100 replicate simulations at that location in the genome.
(EPS)

S45 FigDetailed illustration of Λ statistics and multi-locus genotype frequency spectra in CEU and YRI.(A) Λ plotted in the *LCT* region, vertical dotted lines indicate zoomed region shown in (B) and (C). (B) YRI empirical HFS for 11 windows in the *LCT* region. (C) CEU empirical HFS for 11 windows in the *LCT* region. (D) Λ plotted in the MHC region, vertical dotted lines indicate zoomed region shown in (E) and (F). (E) YRI empirical HFS for 11 windows in the MHC region. (F) CEU empirical HFS for 11 windows in the MHC region. In (B), (C), (E), and (F), numbers above HFS are Λ values for the window rounded to the nearest whole number, and the genome-wide average HFS is highlighted in grey. $q_{i}^{20}$ is the frequency of the *i*th most common MLG truncated to *K* = 20.(EPS)

S46 Fig Maximum Λ observed per window across demography-matched neutral simulations versus recombination rate. For the (A) CEU and (B) YRI populations.
(EPS)

S1 Table Λ statistic thresholds for TGP analyses as calculated from demography-matched neutral simulations. (PDF)

S2 Table Gene ontology enrichment analysis of regions with extreme Λ values in the European (CEU) human population. (PDF)

S3 Table Gene ontology enrichment analysis of regions with extreme Λ values in the African (YRI) human population. (PDF)

S4 Table Spearman correlations of Λ statistics calculated with different distance metrics. From demography-matched neutral whole genome simulations with variable recombination rate (mean across 100 replicates) and from empirical data.
(PDF)

S5 TableRegions of extreme Λ values (unphased analysis) in the CEU population and the genes contained therein.

$\hat{m}$ is the inferred number of sweeping haplotypes, and ${\text{log}}_{10}\left(\hat{A}\right)$ is the estimated sweep width.(PDF)

S6 TableRegions of extreme Λ values (unphased analysis) in the YRI population and the genes contained therein.

$\hat{m}$ is the inferred number of sweeping haplotypes, and ${\text{log}}_{10}\left(\hat{A}\right)$ is the estimated sweep width.(PDF)

S7 TableRegions of extreme Λ values in the New York City rat population that contain annotated genes in genome build RN5.

$\hat{m}$ is the inferred number of sweeping haplotypes, and ${\text{log}}_{10}\left(\hat{A}\right)$ is the estimated sweep width.(PDF)

S8 TableRegions of extreme Λ values in the New York City rat population that do not contain annotated genes in genome build RN5.

$\hat{m}$ is the inferred number of sweeping haplotypes, and ${\text{log}}_{10}\left(\hat{A}\right)$ is the estimated sweep width.(PDF)
