# Supplementary material for: A spatially aware likelihood test to detect sweeps from haplotype distributions
Source: PLoS Genet. 2022 Apr 11;18(4):e1010134. doi: 10.1371/journal.pgen.1010134 (PMC9022890; doi:10.1371/journal.pgen.1010134)
Supplement: S1 Table — (PDF) [file pgen.1010134.s047.pdf]

Population	Phased	Top-1% Λ	Top-0.1% Λ	Max Λ
CEU	Yes	100.464	220.170	992.484
CEU	No	43.444	96.525.547	425.335
YRI	Yes	46.092	105.728	503.981
YRI	No	19.339	38.000	145.126
