# Supplementary material for: A spatially aware likelihood test to detect sweeps from haplotype distributions
Source: PLoS Genet. 2022 Apr 11;18(4):e1010134. doi: 10.1371/journal.pgen.1010134 (PMC9022890; doi:10.1371/journal.pgen.1010134)
Supplement: S2 Table — (PDF) [file pgen.1010134.s048.pdf]

GO molecular function	Fold Enrichment	Raw P-value	FDR
MHC class II receptor activity	> 100	4.05×10^{-15}	6.59×10^{-12}
Immune receptor activity	40.58	6.92×10^{-9}	4.22×10^{-6}
CD8 receptor binding	> 100	1.48×10^{-5}	7.22×10^{-3}
T cell receptor binding	> 100	3.29×10^{-7}	1.78×10^{-4}
Protein-containing complex binding	5.96	2.72×10^{-5}	1.21×10^{-2}
MHC class II protein complex binding	> 100	1.96×10^{-15}	4.79×10^{-12}
MHC protein complex binding	> 100	1.17×10^{-14}	1.43×10^{-11}
Beta-2-microglobulin binding	> 100	4.43×10^{-5}	1.80×10^{-2}
Peptide antigen binding	> 100	4.37×10^{-17}	2.13×10^{-13}
Peptide binding	24.29	6.89×10^{-10}	5.60×10^{-7}
Amide binding	19.57	3.64×10^{-9}	2.54×10^{-6}
Antigen binding	41.08	1.20×10^{-11}	1.17×10^{-8}
