# Supplementary material for: A spatially aware likelihood test to detect sweeps from haplotype distributions
Source: PLoS Genet. 2022 Apr 11;18(4):e1010134. doi: 10.1371/journal.pgen.1010134 (PMC9022890; doi:10.1371/journal.pgen.1010134)
Supplement: S3 Table — (PDF) [file pgen.1010134.s049.pdf]

GO molecular function	Fold Enrichment	Raw P-value	FDR
MHC class II receptor activity	> 100	6.38×10^{-17}	1.04×10^{-13}
Immune receptor activity	25.82	9.27×10^{-12}	9.05×10^{-9}
MHC class II protein complex binding	> 100	2.49×10^{-18}	1.22×10^{-14}
MHC protein complex binding	> 100	2.86×10^{-17}	6.99×10^{-14}
Protein-containing complex binding	3.70	3.52×10^{-5}	1.91×10^{-2}
Peptide antigen binding	93.61	2.79×10^{-15}	3.40×10^{-12}
Peptide binding	10.43	2.14×10^{-7}	1.49×10^{-4}
Amide binding	8.40	1.25×10^{-6}	7.60×10^{-4}
Antigen binding	17.64	2.71×10^{-9}	2.20×10^{-6}
