# Supplementary material for: A spatially aware likelihood test to detect sweeps from haplotype distributions
Source: PLoS Genet. 2022 Apr 11;18(4):e1010134. doi: 10.1371/journal.pgen.1010134 (PMC9022890; doi:10.1371/journal.pgen.1010134)
Supplement: S4 Table — From demography-matched neutral whole genome simulations with variable recombination rate (mean across 100 replicates) and from empirical data. (PDF) [file pgen.1010134.s050.pdf]

Population	Data Type	# windows vs. bps	cM vs. bps
CEU	Simulated	0.929	0.912
YRI	Simulated	0.967	0.895
CEU	Empirical	0.977	0.907
YRI	Empirical	0.967	0.909
