# Supplementary material for: A spatially aware likelihood test to detect sweeps from haplotype distributions
Source: PLoS Genet. 2022 Apr 11;18(4):e1010134. doi: 10.1371/journal.pgen.1010134 (PMC9022890; doi:10.1371/journal.pgen.1010134)
Supplement: S5 Table — m^ is the inferred number of sweeping haplotypes, and log10(A^) is the estimated sweep width. (PDF) [file pgen.1010134.s051.pdf]

Chr	Start (bp)	Stop (bp)	$\widehat{m}$	$\log_{10}(\widehat{A})$	Max Λ	Genes
2	135,517,503	135,699,618	2	8.686	464.366	*ACMSD*, *MIR5590*, *CCNT2-AS1*, *CCNT2*
2	135,699,619	135,889,801	2	8.686	469.480	*CCNT2*, *MAP3K19*, *RAB3GAP1*
2	135,936,894	136,318,298	1	8.686	677.751	*ZRANB3*, *R3HDM1*
2	136,494,985	136,788,904	1	8.686	890.887	*UBXN4*, *LCT*, *LOC100507600*, *MCM6*, *DARS*, *DARS-AS1*
4	34,363,017	34,399,713	4	8.686	440.658	–
4	61,210,873	61,273,068	5	8.686	448.738	–
6	32,491,945	32,694,523	7	7.817	699.735	*HLA-DRB5*, *HLA-DRB6*, *HLA-DRB1*, *HLA-DQA1*, *HLA-DQB1*, *HLA-DQB1-AS1*
