# Supplementary material for: A spatially aware likelihood test to detect sweeps from haplotype distributions
Source: PLoS Genet. 2022 Apr 11;18(4):e1010134. doi: 10.1371/journal.pgen.1010134 (PMC9022890; doi:10.1371/journal.pgen.1010134)
Supplement: S6 Table — m^ is the inferred number of sweeping haplotypes, and log10(A^) is the estimated sweep width. (PDF) [file pgen.1010134.s052.pdf]

Chr	Start (bp)	Stop (bp)	$\widehat{m}$	$\log_{10}(\widehat{A})$	Max Λ	Genes
1	196,838,929	196,882,435	9	8.252	154.62	*CFHR4*
1	223,285,200	223,386,011	5	8.252	197.548	*TLR5*
3	27,139,533	27,182,014	8	8.252	154.481	*NEK10*
3	46,040,267	46,365,291	6	8.252	348.033	*XCR1*, *CCR1*, *CCR3*
3	162,438,975	162,692,695	8	8.252	296.913	–
3	163,802,717	163,857,923	8	8.252	174.977	–
4	12,461,207	12,479,264	8	8.252	151.189	–
4	12,503,155	12,570,561	9	8.252	168.923	–
4	46,843,639	46,884,507	9	8.252	145.785	*COX7B2*
4	74,379,014	74,493,240	8	8.252	160.766	*LOC728040*, *RASSF6*
6	31,171,915	31,401,004	9	7.817	352.419	*HLA-C*, *HLA-B*, *MIR6891*, *MICA*
6	32,510,827	32,635,225	9	7.817	248.927	*HLA-DRB6*, *HLA-DRB1*, *HLA-DQA1*, *HLA-DQB1*, *HLA-DQB1-AS1*
6	32,649,806	32,694,318	10	7.817	180.873	–
6	32,978,997	33,195,684	7	7.817	324.768	*HLA-DPA1*, *HLA-DPB1*, *HLA-DPB2*, *COL11A2*, *RXRB*, *SLC39A7*, *HSD17B8*, *MIR219A1*, *RING1*
6	130,541,399	130,674,414	7	7.817	244.991	*SAMD3*
7	80,186,004	80,428,489	6	8.252	207.636	*CD36*, *SEMA3C*
8	5,767,335	5,824,312	10	8.252	165.733	–
8	9,453,656	9,697,983	8	8.252	176.026	*TNKS*
8	50,022,640	50,229,742	8	8.252	275.713	–
9	11,774,095	11,867,257	9	8.252	161.915	–
9	11,898,626	11,940,738	7	8.252	166.824	–
10	102,043,692	102,310,074	6	8.252	327.657	*BLOC1S2*, *PKD2L1*, *SCD*, *OLMALINC*, *WNT8B*, *SEC31B*, *NDUFB8*, *HIF1AN*
11	42,559,011	42,612,433	8	8.252	152.469	–
11	42,626,446	42,711,242	8	8.252	187.084	–
12	79,543,414	79,794,477	6	8.252	334.766	*SYT1*
12	82,748,538	82,820,129	9	8.252	173.956	*CCDC59*, *METTL25*
12	82,859,617	82,931,718	8	8.252	151.183	*METTL25*
13	89,192,255	89,235,146	9	8.252	157.25	*LINC00433*
14	48,245,871	48,321,935	8	7.817	168.862	*LINC00648*
14	48,355,469	48,392,903	7	7.817	151.657	–
14	48,656,257	48,694,596	9	7.817	156.422	–
14	106,434,952	106,471,410	5	7.817	155.709	*ADAM6*
15	55,111,308	55,359,448	7	8.252	302.699	–
17	3,530,561	3,658,973	6	8.252	294.719	*SHPK*, *CTNS*, *TAX1BP3*, *P2RX5-TAX1BP3*, *EMC6*, *P2RX5*, *ITGAE*, *GSG2*
17	60,593,430	60,624,708	6	8.252	152.548	*TLK2*
19	38,860,718	38,951,198	8	8.252	182.638	*CATSPERG*, *PSMD8*, *GGN*, *SPRED3*, *FAM98C*, *RASGRP4*, *RYR1*
20	37,313,016	37,490,473	7	8.686	159.738	*SLC32A1*, *ACTR5*, *PPP1R16B*
22	36,571,575	36,749,069	7	8.252	184.972	*APOL4*, *APOL2*, *APOL1*, *MYH9*, *MIR6819*
