# Supplementary material for: A spatially aware likelihood test to detect sweeps from haplotype distributions
Source: PLoS Genet. 2022 Apr 11;18(4):e1010134. doi: 10.1371/journal.pgen.1010134 (PMC9022890; doi:10.1371/journal.pgen.1010134)
Supplement: S7 Table — m^ is the inferred number of sweeping haplotypes, and log10(A^) is the estimated sweep width. (PDF) [file pgen.1010134.s053.pdf]

Chr	Start (bp)	Stop (bp)	$\widehat{m}$	$\log_{10}(\widehat{A})$	Max Λ	Genes
1	156,312,419	157,034,325	3	7.765	673.695	*Olr23*, *Olr24*, *Olr25*, *Olr27*, *Olr29*, *Olr30*, *Olr32*, *Olr34*, *Folh1*
1	230,123,826	230,327,101	3	7.765	177.137	*Slc22a24*
2	247,180,473	247,304,944	1	7.727	113.896	*Ndst3*
2	269,229,545	269,542,892	1	7.727	675.289	*Sh3glb1*, *Clca2*, *Clca4l*, *Clca4*, *Clca1*, *Clca5*, *Odf2l*
3	10,383,050	10,578,622	3	7.766	168.801	*Abo3*
4	41,336,974	41,611,989	1	7.910	168.115	*Foxp2*
4	133,838,379	133,973,032	3	7.910	107.921	*Moxd2*, *Prss58*, *Tryx5*
4	211,484,812	211,533,858	4	7.910	93.3354	*Clec2e*
4	216,573,081	216,659,618	2	7.910	106.382	*Cacna1c*
5	107,548,396	107,587,543	1	8.226	92.9657	*Sh3gl2*
6	92,808,416	92,850,870	3	7.837	89.5456	*Lrfn5*
7	17,187,521	17,434,428	1	7.751	147.23	*Vom2r55*
7	27,437,241	27,591,701	2	7.751	130.023	*Nt5dc3*, *Stab2*
7	29,959,185	30,172,142	1	7.751	126.808	*Ano4*, *Nr1h4*
7	45,385,763	45,446,200	2	7.751	93.5232	*Slc6a15*
8	119,571,436	119,673,192	1	8.378	124.087	*Arpp21*
9	55,257,470	56,770,741	1	8.134	301.09	*Tmeff2*
13	1,901,678	2,824,028	2	7.949	410.203	*Dsel*
13	12,195,969	12,424,761	2	7.949	199.78	*Cntnap5c*
13	68,568,273	68,885,107	3	7.949	508.191	*Brinp3*
14	32,656,671	32,892,843	1	7.740	204.445	*Igfbp7*, *Polr2b*
17	80,051,550	80,219,722	2	7.860	103.731	*Fam107b*
