# Supplementary material for: A spatially aware likelihood test to detect sweeps from haplotype distributions
Source: PLoS Genet. 2022 Apr 11;18(4):e1010134. doi: 10.1371/journal.pgen.1010134 (PMC9022890; doi:10.1371/journal.pgen.1010134)
Supplement: S8 Table — m^ is the inferred number of sweeping haplotypes, and log10(A^) is the estimated sweep width. (PDF) [file pgen.1010134.s054.pdf]

Chr	Start (bp)	Stop (bp)	$\widehat{m}$	$\log_{10}(\widehat{A})$	Max Λ	Genes
2	96,271,808	96,560,663	2	7.727	410.932	–
2	96,881,372	97,074,854	3	7.727	326.032	–
2	170,378,708	170,519,263	2	7.727	95.1473	–
2	272,083,254	272,359,881	1	7.727	241.38	–
3	23,325,877	23,538,235	2	7.766	213.939	–
3	143,427,703	143,448,409	1	7.766	90.6533	–
4	40,399,215	40,462,778	1	7.910	97.1179	–
4	47,317,126	47,413,412	3	7.910	110.665	–
4	47,479,582	47,521,098	3	7.910	91.0695	–
5	23,200,838	23,418,591	3	8.226	164.644	–
5	136,100,860	136,251,275	2	8.226	103.145	–
5	136,259,254	136,263,345	2	8.226	92.3248	–
5	136,271,400	136,321,452	2	8.226	104.762	–
5	136,904,389	136,961,220	1	8.226	99.4727	–
5	137,107,962	137,167,762	1	8.226	91.6863	–
6	93,246,089	93,895,837	2	7.837	285.452	–
6	128,257,859	128,342,359	1	7.837	101.541	–
6	151,455,228	151,704,771	1	7.837	394.659	–
6	151,877,456	151,898,077	3	7.837	94.741	–
7	19,461,789	19,650,647	3	7.751	133.615	–
7	43,024,984	43,049,245	2	7.751	89.1577	–
7	43,095,862	43,141,691	2	7.751	106.754	–
7	89,090,760	89,282,665	2	7.751	142.529	–
7	109,432,422	109,528,692	1	7.751	91.6154	–
7	127,786,183	127,890,186	1	7.751	134.217	–
7	137,032,536	137,122,391	1	7.751	120.846	–
8	3,092,342	3,236,525	3	8.378	113.975	–
9	56,797,610	56,900,761	2	8.134	125.534	–
9	57,116,060	57,176,933	1	8.134	93.6	–
9	119,697,786	119,952,597	1	8.134	192.989	–
10	28,182,853	28,209,022	2	8.240	88.2872	–
13	3,073,604	3,247,275	3	7.949	185.906	–
13	13,376,314	13,603,477	1	7.949	185.505	–
13	14,872,399	14,887,445	3	7.949	88.3306	–
13	43,647,576	43,944,224	4	7.949	235.378	–
14	20,095,568	20,119,479	2	7.740	88.9939	–
14	30,706,707	30,902,046	1	7.740	166.608	–
14	30,925,938	30,979,611	1	7.740	95.0194	–
14	30,990,277	31,067,039	2	7.740	95.3088	–
14	35,268,129	35,338,455	1	7.740	91.7566	–
14	67,880,756	68,116,102	1	7.740	169.303	–
14	95,349,113	95,473,128	2	4.103	135.307	–
15	57,136,691	57,510,633	1	7.968	155.564	–
17	79,592,898	79,717,397	1	7.860	116.18	–
18	61,104,703	61,227,007	1	8.141	123.9	–
18	61,609,058	61,788,832	1	8.141	138.145	–
20	54,099,523	54,274,713	3	7.885	118.206	–
